# Attraction in Action: Reduction of Water to Dihydrogen Using Surface-Functionalized TiO_2_ Nanoparticles

**DOI:** 10.3390/nano12050789

**Published:** 2022-02-25

**Authors:** Sven A. Freimann, Catherine E. Housecroft, Edwin C. Constable

**Affiliations:** Department of Chemistry, University of Basel, Mattenstrasse 24a, BPR 1096, 4058 Basel, Switzerland; s.freimann@unibas.ch (S.A.F.); catherine.housecroft@unibas.ch (C.E.H.)

**Keywords:** nanoparticles, anchored-catalyst, heterogeneous catalysis, water reduction

## Abstract

The reactivity of a heterogeneous rhodium(III) and ruthenium(II) complex-functionalized TiO_2_ nanoparticle (NP) system is reported. The ruthenium and rhodium metal complexes work in tandem on the TiO_2_ NPs surface to generate H_2_ through water reduction under simulated and normal sunlight irradiation. The functionalized TiO_2_ NPs outperformed previously reported homogeneous systems in turnover number (TON) and frequency (TOF). The influence of individual components within the system, such as pH, additive, and catalyst, were tested. The NP material was characterized using TGA-MS, ^1^H NMR spectroscopy, FTIR spectroscopy, solid absorption spectroscopy, and ICP-MS. Gas chromatography was used to determine the reaction kinetics and recyclability of the NP-supported photocatalyst.

## 1. Introduction

The increasing energy demand of the world population has led to the unsustainable consumption of fossil fuels and emission of greenhouse gases [1,2,3]. Within the next century, fossil fuels will be substantially depleted if consumption rates or energy sources do not change [4]. The development of cleaner, renewable, available, and less-expensive energy solutions has become a central societal and research imperative [5].

Dihydrogen is an outstanding candidate as fuel, possessing key advantages, including long term storage and carbon-free combustion. Liquid or high-pressure gaseous dihydrogen has high gravimetric energy density while lacking volumetric energy density compared to liquid fossil fuels [6]. Hydrogen is the most abundant element in the universe and the tenth most abundant element in the earth’s crust by weight percentage. Terrestrially, only small amounts of elemental H_2_ occur, with most hydrogen found within molecules, most commonly water [7]. This means dihydrogen for use as fuel must be generated through chemical transformations.

The most common processes for large-scale dihydrogen preparation are water electrolysis and steam methane reforming [8]. Both technologies require high amounts of energy and work best with non-sustainable metal catalysts [8,9,10]. An imperative is improving energy efficiency and using sustainable, recyclable, or easily recoverable catalysts [11]. One option is to combine energy harvesting and dihydrogen evolution using photocatalysts that work under sunlight irradiation [12].

Homogeneous photocatalytic systems can be very efficient, although catalyst recovery can be extremely challenging and cost or energy-intensive [13,14]. In particular, multicomponent systems are problematic. Heterogeneous catalysts are often easier to recover but have the disadvantage of inactive interior volumes with only surface sites being catalytically active [13,14,15]. An alternative is to surface-functionalize nanoparticle (NP) scaffolds composed of cheap and abundant elements with photocatalysts. Such immobilized photocatalysts offer greater catalyst-to-volume ratios than bulk heterogeneous catalysts, which translates to enhanced catalytic activity and turnover. An additional benefit of NPs is the ability to disperse them in liquid phases [16,17,18,19]. A number of heterogeneous NP photocatalytic systems, including CdS NPs [20], Cu-doped TiO_2_ NPs [21] or ZnO NPs [22], Pt-doped TiO_2_ NPs [23] or ZnO NPs [24], Ti^3+^ doped TiO_2_ NPs [25], and TiO_2_ mediated ligand-capped RuO_2_ NPs [26], have been reported as efficient systems for dihydrogen generation. Results of comparable systems are summarized in Table 1.

**Scheme 1:**
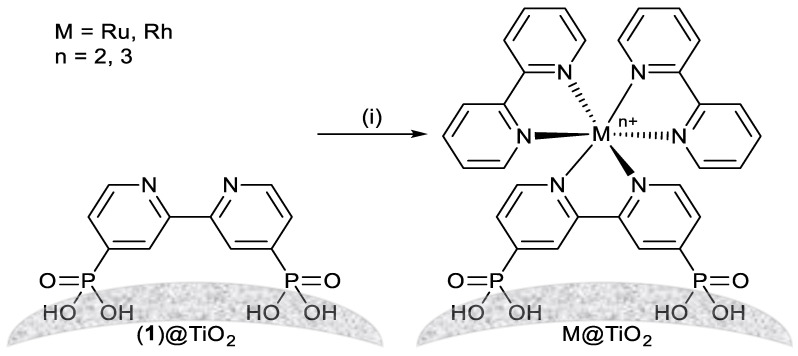
Assembly of metal complexes on TiO_2_ NPs starting from NPs functionalized with **1**. Conditions: (i) MCl_3_·3H_2_O (M = Ru or Rh), bpy, EtOH: H_2_O, 160 °C, autoclave, 1 h.

We now describe the immobilization of components from a previously reported aqueous photochemical system for water reduction under simulated sunlight irradiation [27]. The system utilizes TiO_2_ NPs supporting ruthenium(II) and rhodium(III) 2,2′-bipyridine (bpy) complexes as photosensitizer and relay species, respectively Scheme 1) [27]. The complexes at the desired surface ratio are assembled on [2,2′-bipyridine]-4,4′-diylbis(phosphonic acid) (**1**) functionalized NPs (**1**@TiO_2_). Comparative studies with ZrO_2_ NPs are also reported. ZrO_2_ is an insulator and the NPs are commercially available. The change from TiO_2_ (band gap = 3.2 eV) [28] to ZrO_2_ (band gap = 5.1 eV) [29] was expected to yield insights into metal complex to NP surface bonding and electronic interactions.

The NPs were characterized using Fourier-transform infrared (FTIR) and solid-state absorption spectroscopies, thermogravimetric analysis mass spectrometry (TGA-MS), triple quadrupole inductively coupled plasma mass spectrometry (ICP-QQQ-MS), and matrix-assisted laser desorption/ionization (MALDI) mass spectrometry while dihydrogen generation was analysed using gas chromatography (GC, see ESI† for details).

## 2. Materials and Methods

### 2.1. General

RuCl_3_·3H_2_O was purchased from Oxkem Ltd., Reading, UK. RhCl_3_·3H_2_O was purchased from Johnson Matthey, Materials Technology UK. 2,2′-Bipyridine and triethanolamine (TEOA) were purchased from Apollo Scientific Ltd., Stockport, UK and Sigma-Aldrich Chemie GmbH, Buchs, Switzerland respectively while K_2_[PtCl_4_] was purchased from Alfa Aesar GmbH & Co KG, Karlsruhe, Germany. TiO_2_ NPs (AEROXIDE TiO_2_ P25) were purchased from Evonik Industries, Essen, Germany or Sigma-Aldrich Chemie GmbH, Buchs, Switzerland. Pristine ZrO_2_ NPs (<100 nm particle size) were purchased from Sigma-Aldrich Chemie GmbH, Buchs, Switzerland. For further characterization see ESI†. *cis*-[Ru(bpy)_2_Cl_2_] and anchoring ligand (**1**) were prepared according to the literature (see ESI† for synthetic details) [30,31,32,33,34,35]. Instrumentation details are given in the ESI†. Calculated major MALDI peaks given in the experimental sections were calculated using the most abundant isotopes (e.g., ^102^Ru, ^35^Cl).

### 2.2. Synthetic Procedures

#### 2.2.1. TiO_2_ NPs Functionalization

NP activation and functionalization with ligand (**1**) were carried out according to our previously published procedure [36,37]. The procedures were adjusted according to the molecular weight for anchoring ligand (**1**) (see following section).

#### 2.2.2. Activation of Commercial P25 TiO_2_ NPs

The commercially available NPs were activated as previously reported [36]. The procedure was scaled up as follows. Commercial P25 TiO_2_ NPs (5.00 g) were dispersed by sonication for 15 min in dilute aqueous HNO_3_ (70 mL, 3 M). The mixture was then stirred for 30 min. The suspension was centrifuged (10 min, 7000 rpm) and the NPs were washed once with milliQ water (70 mL). The NPs were added to milliQ water (50 mL) and dispersed by sonication for 10 min. The suspension was then stirred overnight. The suspension was centrifuged (10 min, 7000 rpm) and the NPs were washed with milliQ water (2 × 50 mL). The activated NPs (4.83 g) were stored in a sealed vial under N_2_ after drying under high vacuum. TGA: weight loss/%, 1.8 (<380 °C), 0.3 (380–900 °C). TGA-MS: amu, 18, 30 (<380 °C), 18, 44 (380–900 °C). FTIR spectroscopy: 1607, 1582, 1427, and 1298 cm^−1^. ICP-MS: No ruthenium or rhodium were detected in either the pristine or activated NPs.

#### 2.2.3. Preparation of 1@TiO_2_

The functionalization was performed as previously reported [36,37] with the procedure adjusted for the anchoring ligand 4,4′-bis(phosphonato)-2,2′-bipyridine as follows. (**1**) (25.0 mg, 0.079 mmol, 1 eq.) and milliQ water (18 mL) were added to a microwave vial and dispersed by sonication for 1 min. Activated TiO_2_ NPs (727 mg, 33.9 TiO_2_ eq.) were added. The suspension was dispersed by sonication for 10 min. The microwave vial was sealed, and the reaction mixture heated for 3 h at 130 °C in the microwave reactor. The suspension was centrifuged (20 min, 7000 rpm) after cooling to room temperature. The NPs were separated from the solvent. The white **1**@TiO_2_ NPs (742 mg) were stored in a sealed vial under N_2_ after drying under high vacuum. For NMR spectroscopic measurements, **1**@TiO_2_ NPs (5–10 mg) were dispersed in 500 µL D_2_O in an NMR tube. TGA: weight loss/%, 0.7 (<380 °C), 2.9 (380–900 °C). TGA-MS: amu, 18 (<380 °C), 18, 44 (380–900 °C). FTIR spectroscopy: 1630, 1590, 1540, 1500, 1480, 1430 and 1160 cm^–1^. ICP-MS: No ruthenium or rhodium was detected. Solid-state absorption spectroscopy: 400–670 nm (weak). MALDI *m/z*: 317.1 [(**1**) + H]^+^ (calc. 317.0), 379.1 [(**1**) − H + TiO]^+^ (calc. 378.9), 445.0 [(**1**) + H + Ti_2_O_2_]^+^ (calc. 444.9) and 656.0 [(**1**)_2_ + H + Na]^+^ (calc. 656.0).

#### 2.2.4. Ru@TiO_2_

The metal complex was formed directly on the NP surface. Hence, **1**@TiO_2_ (45.9 mg), RuCl_3_·3H_2_O (1.03 mg, 3.9 µmol), and 2,2′-bipyridine (1.56 mg, 10.0 µmol) were added to a vial. H_2_O (5 mL) and EtOH (3 mL) were added, and the mixture was thoroughly dispersed using sonication and stirring. The suspension was transferred to an autoclave PTFE liner with additional EtOH (2 mL). The autoclave was sealed and then heated in an oven with 320 °C/h to 160 °C. The autoclave was left at 160 °C for 1 h. After cooling down the autoclave was opened and the suspension was centrifuged (20 min, 7000 rpm). The resulting NPs were washed with H_2_O (3 × 10 mL) and EtOH (1 × 10 mL). Ru@TiO_2_ was isolated as dark orange powder. ^1^H-NMR spectroscopy, MALDI, TGA-MS, FTIR spectroscopy, ICP-MS, and solid absorption spectroscopy were performed on the NPs. TGA: weight loss/%, 1.5 (<380 °C), 4.6 (380–900 °C). TGA-MS: amu, 18 (<380 °C), 18, 44 (380–900 °C). FTIR spectroscopy: 1640, 1604, 1465, 1447, 1423, 1398, 1156, and 1049 cm^–1^. ICP-MS: ruthenium present. Solid-state absorption spectroscopy: 400–490 nm, 490–700 nm (weak). MALDI *m/z*: 414.1 [Ru(bpy)_2_]^+^ (calc. 414.0), 535.1 [Ru(**1**) + TiO_2_ + K − 2 H]^+^ (calc. 534.8), 570.1 [Ru(**1**)(bpy) − 4 H]^+^ (calc. 569.9), 602.1 [Ru(bpy)_2_ + CHCA − H]^+^ (calc. 602.1), 728.9 [Ru(**1**)(bpy)_2_ − H]^+^ (calc. 729.0). (CHCA = α-cyano-4-hydroxycinnamic acid).

#### 2.2.5. Ru@TiO_2_ Using [Ru(bpy)_2_Cl_2_]

**1**@TiO_2_ (45.9 mg) and [Ru(bpy)_2_Cl_2_] (3.05 mg, 5.0 µmol) were added to a vial. H_2_O (5 mL) and EtOH (3 mL) were added, and the mixture was thoroughly dispersed using sonication and stirring. The suspension was transferred to an autoclave PTFE liner with additional EtOH (2 mL). The autoclave was sealed and then heated in an oven at a rate of 320 °C/h to 160 °C. The autoclave was left at 160 °C for 1 h. After cooling down, the autoclave was opened and the suspension was centrifuged (20 min, 7000 rpm). The resulting NPs were washed with H_2_O (3 × 10 mL) and EtOH (1 × 10 mL). Ru@TiO_2_ was isolated as an orange powder. ^1^H-NMR spectroscopy, MALDI, TGA-MS, FTIR and solid absorption spectroscopy were performed on the NPs. TGA: weight loss/%, 0.6 (<380 °C), 3.3 (380–900 °C). TGA-MS: amu, 18 (<380 °C), 18, 44 (380–900 °C). FTIR spectroscopy: 1626, 1591, 1544, 1465, 1447, 1428, 1376 and 1155 cm^–1^. Solid-state absorption spectroscopy: 400–490 nm, 490–700 nm (weak). MALDI *m/z*: 414.0 [Ru(bpy)_2_]^+^ (calc. 414.0), 535.0 [Ru(**1**) + TiO_2_ + K – 2 H]^+^ (calc. 534.8), 570.1 [Ru(**1**)(bpy) – 4 H]^+^ (calc. 569.9), 602.0 [Ru(bpy)_2_ + CHCA – H]^+^ (calc. 602.1).

#### 2.2.6. Rh@TiO_2_

The metal complex was formed directly on the NP surface. **1**@TiO_2_ (200 mg), RhCl_3_·3H_2_O (4.51 mg, 17.1 µmol) and 2,2′-bipyridine (6.81 mg, 4.36 µmol) were added to a vial. H_2_O (5 mL) and EtOH (3 mL) were added, and the mixture was thoroughly dispersed using sonication and stirring. The suspension was transferred to an autoclave PTFE liner with additional EtOH (2 mL). The autoclave was sealed and then heated in an oven with 320 °C/h to 160 °C. The autoclave was left at 160 °C for 1 h. After cooling down the autoclave was opened and the suspension was centrifuged (20 min, 7000 rpm). The resulting NPs were washed with H_2_O (3 × 10 mL) and EtOH (1 × 10 mL). Rh@TiO_2_ was isolated as white powder. ^1^H-NMR spectroscopy, MALDI, TGA-MS, FTIR spectroscopy, ICP-MS, and solid absorption spectroscopy were performed on the NPs. TGA: weight loss/%, 1.3 (<380 °C), 3.8 (380–900 °C). TGA-MS: amu, 18 (<380 °C), 18, 44 (380–900 °C). FTIR spectroscopy: 1633, 1607, 1544, 1500, 1470, 1453 1401, 1378, and 1153 cm^–1^. ICP-MS: rhodium present. Solid-state absorption spectroscopy: 400–440 nm, 440–700 nm (weak). MALDI *m/z*: 415.0 [Rh(bpy)_2_]^+^ (calc. 415.0), 450.0 [Rh(bpy)_2_ + Cl]^+^ (calc. 450.0), 603.1 [Rh(bpy)_2_ + CHCA − H]^+^ (calc. 603.1) and 656.1 [(**1**)_2_ + H + Na]^+^ (calc. 656.0).

#### 2.2.7. rR-TiO_2_

The metal complex was formed directly on the NP surface. Hence, **1**@TiO_2_ (241 mg), RuCl_3_·3H_2_O (0.24 mg, 0.9 µmol), RhCl_3_.3H_2_O (5.16 mg, 19.6 µmol), and 2,2′-bipyridine (8.08 mg, 51.8 µmol) were added to a vial. H_2_O (5 mL) and EtOH (3 mL) were added, and the mixture was thoroughly dispersed using sonication and stirring. The suspension was transferred to an autoclave PTFE liner with additional EtOH (2 mL). The autoclave was sealed and then heated in an oven at a rate of 320 °C/h to 160 °C. The autoclave was left at 160 °C for 1 h. After cooling down the autoclave was opened and the suspension was centrifuged (20 min, 7000 rpm). The resulting NPs were washed with H_2_O (3 × 10 mL) and EtOH (1 × 10 mL). rR@TiO_2_ was isolated as an orange powder. ^1^H-NMR spectroscopy, MALDI, TGA-MS, FTIR spectroscopy, ICP-MS and solid absorption spectroscopy were performed on the NPs. TGA: weight loss/%, 1.1 (<380 °C), 3.7 (380–900 °C). TGA-MS: amu, 18 (<380 °C), 18, 44 (380–900 °C). FTIR spectroscopy: 1627, 1608, 1546, 1500, 1470, 1453, 1412, 1373 and 1149 cm^–1^. ICP-MS: ruthenium and rhodium present. Solid-state absorption spectroscopy: 400–490 nm, 490–700 nm (weak). MALDI *m/z*: 415.0 [Rh(bpy)_2_]^+^ (calc. 415.0), 450.0 [Rh(bpy)_2_ + Cl]^+^ (calc. 450.0) and 603.1 [Rh(bpy)_2_ + CHCA − H]^+^ (calc. 603.1).

#### 2.2.8. RR-TiO_2_

The metal complex was formed directly on the NP surface. **1**@TiO_2_ (340 mg), RuCl_3_.3H_2_O (2.39 mg, 9.1 µmol), RhCl_3_·3H_2_O (5.16 mg, 19.6 µmol) and 2,2′-bipyridine (11.3 mg, 72.3 µmol) were added to a vial. H_2_O (5 mL) and EtOH (3 mL) were added, and the mixture was thoroughly dispersed using sonication and stirring. The suspension was transferred to an autoclave PTFE liner with additional EtOH (2 mL). The autoclave was sealed and then heated in an oven with 320 °C/h to 160 °C. The autoclave was left at 160 °C for 1 h. After cooling down, the autoclave was opened and the suspension was centrifuged (20 min, 7000 rpm). The resulting NPs were washed with H_2_O (3 × 10 mL) and EtOH (1 × 10 mL). RR-TiO_2_ was isolated as a dark orange powder. ^1^H-NMR spectroscopy, MALDI, TGA-MS, FTIR spectroscopy, ICP-MS and solid absorption spectroscopy were performed on the NPs. TGA: weight loss/%, 1.5 (<380 °C), 3.5 (380–900 °C). TGA-MS: amu, 18 (<380 °C), 18, 44 (380–900 °C). FTIR spectroscopy: 1643, 1542, 1498, 1472, 1447, 1423, 1398, 1375 and 1149 cm^–1^. ICP-MS: ruthenium and rhodium present. Solid-state absorption spectroscopy: 400–490 nm, 490–700 nm (weak). MALDI *m/z*: 415.1 [Rh(bpy)_2_]^+^ (calc. 415.0), 450.1 [Rh(bpy)_2_ + Cl]^+^ (calc. 450.0), 603.1 [Rh(bpy)_2_ + CHCA − H]^+^ (calc. 603.1) and 656.1 [(**1**)_2_ + H + Na]^+^ (calc. 656.0), 729.1 [Ru(**1**)(bpy)_2_ − H]^+^ (calc. 729.0).

#### 2.2.9. Preparation of 1@ZrO_2_

The functionalization was performed as previously reported [36,37] without the acid treatment activation step. The procedure was further adjusted for the anchoring ligand 4,4′-bis(phosphonato)-2,2′-bipyridine and a different NP surface as follows. (**1**) (20.0 mg, 0.063 mmol, 1 eq.) and milliQ water (18 mL) were added to a microwave vial and dispersed by sonication for 1 min. ZrO_2_ NPs (897 mg, 6.9 ZrO_2_ eq.) were added. The suspension was dispersed by sonication for 10 min. The microwave vial was sealed, and the reaction mixture heated for 3 h at 130 °C in the microwave reactor. The suspension was centrifuged (20 min, 7000 rpm) after cooling to room temperature. The NPs were separated from the solvent and washed with H_2_O (3 × 10 mL) and EtOH (1 × 10 mL). The white **1**@ZrO_2_ NPs (856 mg) were stored in a sealed vial under N_2_ after drying under high vacuum. For NMR spectroscopic measurements, **1**@ZrO_2_ NPs (5–10 mg) were dispersed in 500 µL D_2_O in an NMR tube. TGA: weight loss/%, 0.6 (<380 °C), 1.7 (380–900 °C). TGA-MS: amu, 18 (<380 °C), 18, 44 (380–900 °C). FTIR spectroscopy: 1625, 1590, 1542, 1496, 1473, 1432, 1376, 1223, 1152, 1038, 1000, 840, 744, 658, 562 and 480 cm^–1^.

#### 2.2.10. Ru@ZrO_2_

The metal complex was formed directly on the NP surface. Hence, **1**@ZrO_2_ (142 mg), RuCl_3_·3H_2_O (2.07 mg, 7.9 µmol), and 2,2′-bipyridine (2.50 mg, 16.0 µmol) were added to a vial. H_2_O (5 mL) and EtOH (3 mL) were added, and the mixture was thoroughly dispersed using sonication and stirring. The suspension was transferred to an autoclave PTFE liner with additional EtOH (2 mL). The autoclave was sealed and then heated in an oven with 320 °C/h to 160 °C. The autoclave was left at 160 °C for 1 h. After cooling down the autoclave was opened and the suspension was centrifuged (20 min, 7000 rpm). The resulting NPs were washed with H_2_O (3 × 10 mL) and EtOH (1 × 10 mL). Ru@ZrO_2_ was isolated as an ochre powder. ^1^H-NMR spectroscopy, MALDI, TGA-MS and FTIR spectroscopy were performed on the NPs. TGA: weight loss/%, 0.6 (<380 °C), 2.1 (380–900 °C). TGA-MS: amu, 18 (<380 °C), 18, 44 (380–900 °C). FTIR spectroscopy: 1644, 1604, 1544, 1465, 1447, 1422, 1398, 1223, 1160, 1122, 1057, 913, 744, 658, 562, and 480 cm^–1^.

#### 2.2.11. rR-ZrO_2_

The metal complex was formed directly on the NP surface. Hence, **1**@ZrO_2_ (366 mg), RuCl_3_·3H_2_O (0.24 mg, 0.9 µmol), RhCl_3_.3H_2_O (5.16 mg, 19.6 µmol) and 2,2′-bipyridine (6.45 mg, 41.3 µmol) were added to a vial. H_2_O (5 mL) and EtOH (3 mL) were added, and the mixture was thoroughly dispersed using sonication and stirring. The suspension was transferred to an autoclave PTFE liner with additional EtOH (2 mL). The autoclave was sealed and then heated in an oven with 320 °C/h to 160 °C. The autoclave was left at 160 °C for 1 h. After cooling down the autoclave was opened and the suspension was centrifuged (20 min, 7000 rpm). The resulting NPs were washed with H_2_O (3 × 10 mL) and EtOH (1 × 10 mL). rR@ZrO_2_ was isolated as light orange powder. ^1^H-NMR spectroscopy, MALDI, TGA-MS and FTIR spectroscopy were performed on the NPs. TGA: weight loss/%, 0.4 (<380 °C), 2.1 (380–900 °C). TGA-MS: amu, 18 (<380 °C), 18, 44 (380–900 °C). FTIR spectroscopy: 1633, 1606, 1589, 1541, 1498, 1468, 1452, 1429, 1398, 1375, 1215, 1156, 1042, 1000, 910, 839, 744, 658, 562, and 480 cm^–1^.

### 2.3. Dihydrogen Generation

#### 2.3.1. General Procedure

The system for dihydrogen generation used metal complex functionalized NPs [38] as photo- and electrocatalysts, triethanolamine as a sacrificial electron donor, K_2_[PtCl_4_] as catalyst to facilitate dihydrogen formation (possibly by Pt NP formation), bpy as additive, aqueous H_2_SO_4_ for pH adjustment, and milliQ water as solvent. As [Ru(bpy)_3_]^2+^ and [Rh(bpy)_3_]^3+^ are somewhat photolabile under the operating conditions, the additional bpy was added to regenerate ruthenium and rhodium surface-bound complexes. 

In a 5 mL microwave vial TEOA (2.52 mmol, 376 mg), K_2_[PtCl_4_] (1.7 µmol, 0.70 mg) and 2,2′-bipyridine (18.6 µmol, 2.91 mg) were added together with milliQ water and aqueous H_2_SO_4_ (1M) to modify the pH. Experiments performed at pH 10 used no aqueous H_2_SO_4_ (1M) and 6 mL milliQ water while experiments performed at pH 7.5 used 1 mL aqueous H_2_SO_4_ (1M) and 5 mL milliQ water. Metal complex-functionalized NPs were added (114.1 mg). The vial was flushed with nitrogen and then sealed. The suspension was sonicated (10 min) and thoroughly shaken. Nitrogen was bubbled through the suspension for 10 min. The vial was irradiated for 4–8 h at a slight angle (5°) with a sun simulator generating 1200 W m^–2^. The suspension was shaken hourly. Headspace samples for gas chromatography were collected using a syringe and transferred to a 10 mL GC vial for analysis. The measured GC integral was converted to mL of H_2_ with a calibration made by injecting several known volumes of dihydrogen.

#### 2.3.2. Kinetic Measurements

Using the general procedure, a kinetic run was performed at pH 7.5 using rR@TiO_2_ as photocatalyst. Two vials were prepared and the H_2_ evolution was measured hourly by collecting the headspace by syringe and transferring it to a 10 mL GC vial for GC analysis. After each collection, the suspension was bubbled with N_2_ for 5 min and then irradiation was continued. The vials were irradiated in total for 8 h and 9 h respectively.

#### 2.3.3. Recycle Measurements

Using the general procedure, a recycling experiment was performed preparing two vials at pH 7.5 using rR@TiO_2_ as photocatalyst with 4 h irradiation for each cycle. H_2_ evolution was recorded after each cycle by collecting the headspace by syringe and transferring it to a 10 mL GC vial for GC analysis. After each collection, the suspension was centrifuged, the supernatant was removed, the NPs were washed with water (4 × 10 mL). Subsequently, the NPs were dried under high vacuum. Following the general procedure, the recycled NPs were used instead for the next cycle.

## 3. Results and Discussion

### 3.1. Ligand Functionalization, Surface Complexation and Material Characterisation

#### 3.1.1. Anchoring Ligand Functionalization

We commenced by establishing a reliable method for the preparation of **1**@TiO_2_ and its subsequent metallation. Anchoring ligand **1** was prepared according to the literature [31,32,33,34,35] with the phosphonic acid being chosen for stable binding to TiO_2_ surfaces in neutral, slightly basic and slightly acidic conditions [36,37]. The bpy metal-binding domain in **1** is commensurate with the assembly of surface-bound {M(bpy)_3_}^n+^ (M = Ru, *n* = 2; M = Rh, *n* = 3) motifs. The previously developed method [36,37] for NP anchor functionalization with tpy metal-binding domains was adapted for anchoring ligand **1**. In this case, 32.2 eq. of activated NPs [38,39] were dispersed with anchoring ligand **1** in water and heated to 130 °C for 3 h in a microwave reactor (see Experimental section for full details).

The purchased ZrO_2_ NPs were functionalized using similar techniques but without prior activation. The ZrO_2_ NPs had a diameter of 100 nm changing the surface area to volume ratio significantly (from 28% to 6%). The functionalization method was optimized using our previously established formula [38] to give an adjusted ratio of 6.9 ZrO_2_ eq. to 1 eq. anchoring ligand. The resulting NPs were carefully washed to avoid non-bound free ligand.

#### 3.1.2. Ligand Functionalized NP Characterisation Methods

The **1**@TiO_2_ NPs were analysed using TGA-MS (see Figure S1) and showed a small weight loss (<1%) in two steps (<120 °C, <380 °C) attributed to the loss of physisorbed and chemisorbed water. This was further seen in the coupled MS, which showed peaks with *m/z* of 17 and 18 for HO and H_2_O respectively. A further weight loss of 2.9% over the range of 380–900 °C was measured and attributed to the decomposition of the anchoring ligand on the surface. The MS in this range showed the decomposition product of the ligand to be CO_2_. In contrast, activated NPs (see Figure S2) showed almost no weight loss over the range of 380–900 °C. To verify that the organic weight loss within the 380–900 °C range was not caused by non-covalently bound free ligand, the ^1^H NMR spectrum of **1**@TiO_2_ was measured in D_2_O (see Figure S3) and showed no significant amounts of free ligand in solution (ligand bound to the NPs is not observed) [36]. Further characterisation with FTIR spectroscopy (see Figure S4) and solid-state absorption spectroscopy (see Figure S5) revealed peaks at 1630, 1590, 1540, 1500, 1480, 1430, and 1160 cm^–1^ and 400–670 nm respectively which are not observed for pristine activated NPs. The additional peaks found for **1**@TiO_2_ in FTIR spectroscopy did match to the pristine anchoring ligand. MALDI mass spectrometry showed peaks arising from species consistent with surface binding of the phosphonate ligand (See Figures S6–S8).

**1**@ZrO_2_ was characterized similarly to **1**@TiO_2_: TGA-MS, ^1^H NMR spectroscopy, and FTIR spectroscopy (see Figures S9–S12) were measured. **1**@ZrO_2_ showed a weight loss of 1.7% over the range of 380–900 °C, which was attributed to the decomposition of the anchoring ligand on the surface. The MS in this range showed the decomposition product of the ligand to be CO_2_. The commercial NPs did not show any weight loss other than H_2_O. ^1^H NMR spectroscopy in D_2_O was used to verify absence of non-covalently bound free ligand and no significant amounts of free ligand in solution were observed. FTIR spectroscopy showed several weak additional peaks compared to the commercial NPs within the 1600 and 800 cm^–1^ region (see ESI† Figure S12) originating from the anchoring ligand.

#### 3.1.3. Nanoparticle Surface Complexation

Metal complexes were assembled directly on the NP surface using methods derived from literature procedures for heteroleptic ruthenium(II) complexes, replacing free ligand by **1**@TiO_2_ (Table 2) [35]. Orange Ru@TiO_2_ was isolated from the reaction of **1**@TiO_2_ NPs [38], RuCl_3_.3H_2_O, and bpy in 1:1 water-ethanol in an autoclave at 160 °C for 1 h (see Experimental section for full details). Ru@TiO_2_ can also be formed by replacing RuCl_3_.3H_2_O and bpy with [Ru(bpy)_2_Cl_2_], giving a similar product (TGA-MS, ^1^H NMR spectroscopy, FTIR and solid-state absorption spectroscopies, MALDI mass spectrometry see Figures S13–S27) differing only slightly in colour and small weight loss differences during the TGA measurement. All subsequent metal complex-functionalized NPs (Table 2) were prepared in the autoclave with H_2_O/EtOH as solvent using the one-pot (MCl_3_ + bpy) procedure described in the experimental. The abbreviation rR@TiO_2_ describes metal complex functionalized NPs with a small amount of ruthenium vs. rhodium (1:20) on the surface, while for RR@TiO_2_ ruthenium and rhodium surface concentrations are comparatively similar (1:2). The first letter in rR and RR always refers to ruthenium and the second to rhodium.

The metal complexes Ru@ZrO_2_ and rR@ZrO_2_ were prepared using **1**@ZrO_2_ as starting material and followed the procedure described above with TiO_2_.

#### 3.1.4. Complex Functionalized Characterisation Methods

All metal complex-functionalized NPs species were characterized using the same methods as **1**@TiO_2_. For both Ru@TiO_2_ and Rh@TiO_2_, TGA-MS (Figures S13 and S28) confirmed a higher weight loss (380–900 °C) than **1**@TiO_2_. ^1^H NMR spectroscopy (Figures S15 and S29) showed that no labile species were adsorbed on the NPs. In contrast to **1**@TiO_2_, FTIR spectroscopy (Figures S16 and S30) showed absorptions between 1650 and 1590 cm^−1^ in addition to 1540, 1470, 1450, 1400, and 1150 cm^–1^ originating from the organic ligands. Solid-state absorption spectroscopy showed broad and weak absorptions between 400 and 670 nm. Additionally Rh@TiO_2_ showed a more pronounced absorption band at 420 nm (Figure S31), whilst any surface bound ruthenium complex gave dominant absorptions between 410 and 490 nm (Figure S17). The MALDI mass spectrum of Rh@TiO_2_ (see Figures S32–S35) showed masses with the correct isotope pattern at *m/z*: 415.0 [Rh(bpy)_2_]^+^ (calc. 415.0), 450.0 [Rh(bpy)_2_ + Cl]^+^ (calc. 450.0), 603.1 [Rh(bpy)_2_ + CHCA − H]^+^ (calc. 603.1) and 656.1 [(**1**)_2_ + H + Na]^+^ (calc. 656.0), while that of Ru@TiO_2_ (Figures S18–S27) showed masses with the correct isotope patterns at *m/z*: 414.1 [Ru(bpy)_2_]^+^ (calc. 414.0), 535.1 [Ru(**1**) + TiO_2_ + K − 2 H]^+^ (calc. 534.8), 570.1 [Ru(**1**)(bpy) − 4 H]^+^ (calc. 569.9), 602.1 [Ru(bpy)_2_ + CHCA − H]^+^ (calc. 602.1), 728.9 [Ru(**1**)(bpy)_2_ − H]^+^ (calc. 729.0). For NPs containing both ruthenium and rhodium, the observed isotope distribution in the MALDI mass spectrum confirmed that the rhodium complex was dominant (Figures S36–S44). For full characterisation details, see Section 2 or ESI† Figures S36–S49.

**Figure 1:**
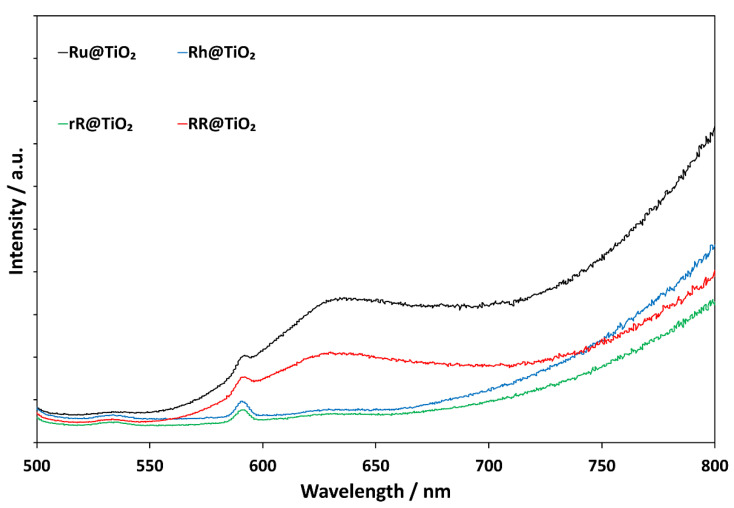
Emission spectra of complex-functionalized NPs after excitation at 450 nm. Excitation slit width 3 nm, emission slit width 10 nm.

Emission spectra of the metal complex functionalized NPs dispersed in water were recorded (Figure 1). The suspension was excited at 450 nm. Ru@TiO_2_ showed a broad emission at 634 nm while RR@TiO_2_ showed a broad emission at 630 nm. Both Rh@TiO_2_ and rR@TiO_2_ were non-emissive.

The NPs were further investigated using ICP-MS (see Table 3). Ruthenium or rhodium functionalized NPs showed their respective elements. It was not possible to perform an exact surface concentration measurement of the elements with ICP-MS as even concentrated nitric acid did not remove all of the surface-bound species from the NP.

For Ru@ZrO_2_ and rR@ZrO_2_, characterization was performed using TGA-MS, ^1^H NMR spectroscopy, and FTIR spectroscopy (see Figure S12 and Figures S50–S52). TGA-MS of Ru@ZrO_2_ and rR@ZrO_2_ revealed a smaller weight loss of 2.1% (380–900 °C) compared to the TiO_2_ equivalents Ru@TiO_2_ (4.6%) and rR@TiO_2_ (3.7%). This was within expectations considering the weight loss seen for **1**@ZrO_2_. Importantly, an increase from **1**@ZrO_2_ to Ru@ZrO_2_ or rR@ZrO_2_ was still observed. ^1^H NMR spectroscopy was used to verify the absence of non-bound anchoring ligand while the FTIR spectra showed only minor differences to **1**@ZrO_2_ within 1600–800 cm^–1^.

### 3.2. Dihydrogen Generation

#### 3.2.1. Performance and Influence of Individual Components during Dihydrogen Generation

Experimental details are given in Section 2.3.1. The study revealed that **1**@TiO_2_, Ru@TiO_2_, and Rh@TiO_2_ all produce H_2_ under irradiation (Table 4) but the gas generation is significantly higher when both ruthenium and rhodium are present on the surface. It is especially interesting that when a single batch of NPs was functionalized with both metal complexes (rR@TiO_2_ or RR@TiO_2_), the H_2_ production was more than double compared to that using an equivalent mixture of Ru@TiO_2_ and Rh@TiO_2_. We propose that the additional efficiency for rR@TiO_2_ and RR@TiO_2_ can be explained by an energy transfer from Ru to Rh which promotes the dihydrogen generation [27,40]. The recorded emission spectra of each species support this proposal as rR@TiO_2_ is not emissive. It has to be noted that the energy transfer must be relatively inefficient since with higher ruthenium concentrations on the surface (RR@TiO_2_ vs. rR@TiO_2_) the metal complex functionalized NPs are emissive again.

Further experiments were performed to test the influence of each component in the system. In the absence of K_2_[PtCl_4_], there was a strong decrease in efficiency while K_2_[PtCl_4_] on its own gave no H_2_ generation. Removing bpy had a less significant influence on the H_2_ generation but slightly lowered the efficiency. In contrast, increasing the bpy concentration had no effect on the efficiency. TEOA was essential for dihydrogen formation. Another important parameter was the pH; at pH 10 the system generated dihydrogen at half the rate observed at pH 7.5. A pH dependence is expected as the formal driving force of the reaction (ΔG) will depend upon pH according to the Nernst equation (ΔG = −nFE) since the key step in the formation of H_2_ involves a proton. A significant improvement in H_2_ formation (17%, TOF_Rh_ = 3.4 × 10^–3^ s^–1^, TOF_Ru_ = 7.4 × 10^–2^ s^–1^) could be observed by simple stirring of the suspension instead of periodic shaking. This shows when the metal complex functionalized NPs settle and block the light, they partially hinder a successful water reduction on covered NPs. For this observation to be possible, a successful surface functionalization must have happened. A further experiment was performed by using normal sunlight, resulting in respectable dihydrogen generation (see Table 4, Entry 15 ^g^), especially since the weather conditions were not bright sunlight.

The dihydrogen generation experiment was expanded using ZrO_2_ NPs as the metal complex carrier material. Since ZrO_2_ is an insulator, the change from TiO_2_ (band gap = 3.2 eV) [28] to ZrO_2_ (band gap = 5.1 eV) [29] was expected to hinder the H_2_ generation during the experiment if the metal complex is surface-bound and interacting electronically with the NP. Hence, the experiment (see Table 4, Entry 16 ^f^) yielding almost no H_2_ generation during the irradiation was interesting and strongly implies mediation by the semiconducting TiO_2_ nanoparticles.

#### 3.2.2. Kinetics of rR@TiO_2_ during Light Irradiation

**Figure 2:**
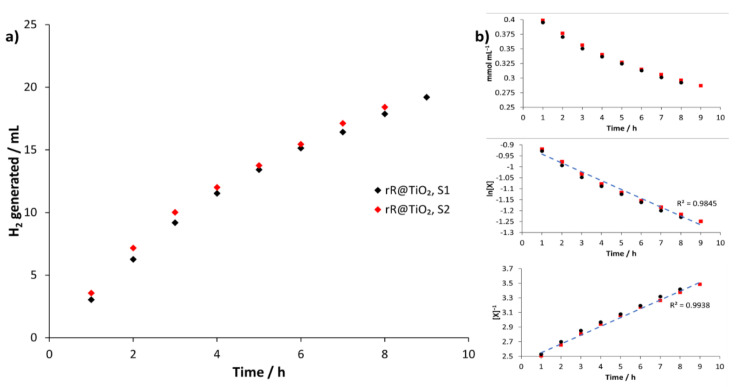
(**a**) Graphical representation of H_2_ generated using rR@TiO_2_ in mL during light irradiation experiments versus time for two samples. (**b**) Zeroth order reaction graph (**top**), concentration (mmol per mL) starting material during kinetic irradiation measurements of two samples (red and black) against time (hours); 1st order reaction graph (**middle**), natural logarithm of starting material concentration [X] during kinetic irradiation measurements of two samples (red and black) against time (hours), linear trendline through datapoints (blue); 2nd order reaction graph (**bottom**), One over concentration [X] of starting material during kinetic irradiation measurements of two samples (red and black) against time (hours), linear trendline through data points (blue).

The kinetics of the H_2_ formation when using rR@TiO_2_ in the general setup was investigated and the experimental results are plotted in Figure 2a) and shown in Table 5. Experimental details are given in Section 2.3.2. Analysis of the data in Figure 2 when TEOA was considered as a reactant (see Figure 2b) indicated first order kinetics (R_2_ = 0.986) or second order kinetics (R_2_ = 0.995). This observation is consistent with the mechanism proposed by Kirch et al. [27]. Using these data, a minimal TON of 86 (TOF = 2.7 × 10^–3^ s^–1^) and TON 1844 (TOF = 5.7 × 10^–2^ s^–1^) can be calculated for the rhodium and ruthenium surface-bound complexes respectively. Furthermore, the amount of H_2_ produced after 9 h irradiation corresponds to a depletion of 32% of TEOA. 

#### 3.2.3. Recyclability of rR@TiO_2_

**Figure 3:**
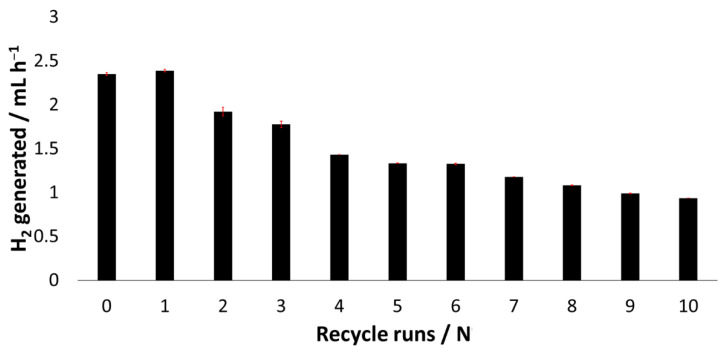
Graphical representation of dihydrogen generated measured through GC analysis after each cycle using rR@TiO_2_ as photocatalyst, standard deviation indicated with red error bars.

Our motivation was to develop a system that could be recycled multiple times and still generate dihydrogen upon irradiation (see Video 1 in the ESI† for visual representation). After an initial run, the NPs were separated from the solution and washed several times with water to ensure the complete removal of the solution components. The NPs were then dried under high vacuum and redispersed in milliQ water at pH 7.5 with fresh TEOA, bpy and K_2_[PtCl_4_]. After each run, the headspace was collected and measured using GC analysis (see Table 6). Overall, rR@TiO_2_ NPs were most suitable for multiple cycles with only a slight decline in efficiency (see Figure 3). We believe that the decline in efficiency is due to the loss of NPs in the recycling processing (10 wt.% after 8 cycles) and defunctionalization of the NP surface. Using the data, a minimal TON of 300 (TOF = 1.9 × 10^–3^ s^–1^) and TON 6424 (TOF = 4.1 × 10^–2^ s^–1^) can be calculated for the rhodium(III) and ruthenium(II) surface bound complexes, respectively.

## 4. Conclusions

We have demonstrated the immobilization of an established photochemical system for the solar generation of dihydrogen using sunlight by binding photo- and redox-active Rh and Ru complexes to TiO_2_ NP surfaces. We further show that binding both metal complexes to the same NP improves the photocatalytic efficiency. The kinetic rate order and recyclability were determined. The NPs could be recycled multiple times and retain dihydrogen generation capacity. TON and TOF of the system were determined and exceeded the previous reported similar homogenous system. The dihydrogen genera ration was monitored using GC while the NPs were characterized using various methods, including MALDI, FTIR spectroscopy, TGA-MS, solid-absorption spectroscopy, fluorescence emission spectroscopy, and ICP-MS.

## Figures and Tables

**Table 1:** Comparative H_2_ evolution materials in the literature.

System	Cat_A_/ µmol	Cat_B_/ µmol	Irr./ h	Yield H_2_/ µmol	TON_A_	TOF_A_/ h^–^^1^	TON_B_	TOF_B_/ h^–^^1^
CdS ^a^ [20]	1730	-	6	283	0.2	0.0	-	-
TiO_2_ p25 ^a^ [20]	3130	-	6	62	0.0	0.0	-	-
0.25%Pt@TiO_2_ ^b^ [23]	1.3	-	3	432	337	112.4	-	-
0.75%Pt@ZnO ^c^ [24]	3.8	-	3	745	194	64.6	-	-
Ru@RuO_2_PPTiO_2_-RuP ^d^ [26]	0.6	-	10	111	176	17.6	-	-
Ru(bpy)_3_^2+^, Rh(bpy)_3_^3+ e^ [27]	1.8	11.7	32	1359	748	23.4	116	3.6

^a^ Using TEOA (0.67 M) as electron donor, ^b^ using TEOA (0.25 M) as electron donor and Eosin Y as dye at 1:83 to photocatalyst, ^c^ using TEOA (0.76 M) as electron donor and Eosin Y as dye at 1:2 to photocatalyst, ^d^ using TEOA (0.2 M) as electron donor, ^e^ using TEOA (0.42 M) as electron donor and K_2_[PtCl_4_] (0.28 mM) as catalyst.

**Table 2:** Different metal complex functionalized nanoparticles.

Entry	1@TiO_2_ ^a^	RuCl_3_	RhCl_3_	bpy
Ru@TiO_2_	1.0 eq.	0.79 eq.	0	2.0 eq.
Rh@TiO_2_	1.0 eq.	0	0.79 eq.	2.0 eq.
rR@TiO_2_	1.0 eq.	0.04 eq.	0.76 eq.	2.0 eq.
RR@TiO_2_	1.0 eq.	0.25 eq.	0.54 eq.	2.0 eq.

^a^ **1**@TiO_2_ equivalents represent estimated amount of **1** on the NP surface.

**Table 3:** ICP-MS concentration and standard deviation of functionalized TiO_2_ NPs.

Sample	^47^Ti Conc. µg/L	^47^Ti Conc. RSD ^a^	^101^Ru Conc. µg/L	^101^Ru Conc. RSD ^a^	^103^Rh Conc. µg/L	^103^Rh Conc. RSD ^a^	^89^Y (ISTD) Conc. µg/L	^89^Y (ISTD) Conc. RSD ^a^
c-NPs	16,756.5	6.1	0.1	17.7	0.1	5.5	145,600.3	4.0
a-NPs	21,460.7	5.6	0.3	127.0	0.3	106.3	150,634.5	4.6
1@TiO_2_	20,492.8	4.7	0.1	12.8	0.1	2.9	146,834.6	5.6
Ru@TiO_2_	21,142.6	21.0	232.7	2.4	0.1	4.0	141,660.1	5.1
Rh@TiO_2_	21,879.5	5.7	0.2	9.6	117.7	3.6	148,959.7	5.3
rR@TiO_2_	20,350.4	2.6	22.2	3.1	89.6	2.5	142,814.2	5.4
RR@TiO_2_	18,659.6	5.9	62.0	4.2	53.5	2.9	146,352.9	5.9
rR@TiO_2_^b^	13,466.9	2.9	3.5	3.2	3.1	4.5	142,412.9	6.5

^a^ Relative standard deviation in percentage, triplicate measurements, ^89^Y used as internal standard to account for matrix effects, ^b^ rR@TiO_2_ after 10 dihydrogen catalytic cycles were measured.

**Table 4:** Performed dihydrogen generating experiments; see Table 2 for NP abbreviations.

Entry No.	NPs /µmol	Byp /µmol	pH	Time /h	GCI ^a^ /a. u.	H_2_ /mL (mL h^−1^)
1	Ru@TiO_2_/1.5	18.6	10	8	152,250	3.14 (0.39)
2	Rh@TiO_2_/9.3	18.6	10	8	199,140	4.11 (0.51)
3 ^b^	Ru@TiO_2_ + Rh@TiO_2_/9.7	18.6	10	8	255,530	5.27 (0.66)
4	rR@TiO_2_/9.7	18.6	10	8	451,170	9.30 (1.16)
5	rR@TiO_2_/9.7	18.6	7.5	4	455,940	9.40 (2.34)
6	rR@TiO_2_/9.7	0	7.5	4	332,280	6.85 (1.71)
7 ^c^	rR@TiO_2_/9.7	18.6	7.5	4	34,720	0.50 (0.13)
8	RR@TiO_2_/9.7	18.6	7.5	4	384,200	7.92 (1.98)
9 ^d^	RR@TiO_2_/13.0	18.6	7.5	4	398,860	8.23 (2.06)
10 ^d^	RR@TiO_2_/13.0	210	7.5	4	397,710	8.20 (2.05)
11 ^e^	a-NP	18.6	7.5	4	138,600	2.86 (0.71)
12	1@TiO_2_/12.2	18.6	7.5	4	134,050	2.76 (0.69)
13	0	18.6	7.5	4	0	0.00 (0.00)
14 ^f^	rR@TiO_2_/9.7	18.6	7.5	4	533,930	11.0 (2.75)
15 ^g^	rR@TiO_2_/9.7	18.6	7.5	4	318,210	6.5 (1.64)
16 ^f^	rR@ZrO_2_/9.7	18.6	7.5	4	29,720	0.6 (0.15)
17 ^h^	rR@TiO_2_/9.7	0	7.5	4	88,360	1.82 (0.46)

^a^ GC integral (GCI) was adjusted for pre-existing nitrogen headspace in the reaction vial and partial sampling during the GC measurement, ^b^ using Ru@TiO_2_ (5.1 mg) and Rh@TiO_2_ (109 mg), ^c^ no K_2_[PtCl_4_] used, ^d^ vial headspace was measured after 2 h and 4 h irradiation, generated H_2_ was added together, ^e^ 114.1 mg a-NP were used, ^f^ vial was stirred instead of shaken hourly, ^g^ vial was irradiated using normal sunlight instead, ^h^ using 18.6 mmol phen as additive.

**Table 5:** Performed hourly kinetic dihydrogen generating experiment of two sample vials.

Runtime/h	GC Integral ^a^ S1 /a. u.	H_2_ Generated S1 /mL	GC Integral ^a^ S2 /a. u.	H_2_ Generated S2 /mL
1	146,050	3.04	171,190	3.57
2	154,530	3.22	172,650	3.60
3	143,060	2.92	136,850	2.85
4	112,380	2.34	95,670	1.99
5	90,960	1.89	83,770	1.75
6	82,450	1.72	81,480	1.70
7	61,900	1.29	80,410	1.67
8	69,260	1.44	62,050	1.29
9	52,740	1.33	-	-

^a^ GC integrals were adjusted for pre-existing nitrogen headspace in the reaction vial and partial sampling during the GC measurement.

**Table 6:** Performed recycle dihydrogen generating experiments of two sample vials.

Cycle/N	GC Integral ^a^/a. u.	NPs Runtime/h	H_2_/mL (mL h^−1^)
0	455,940	4	9.40 (2.35)
1	463,320	8	9.55 (2.38)
2	372,850	12	7.68 (1.92)
3	344,720	16	7.10 (1.77)
4	277,740	20	5.73 (1.43)
5	258,630	24	5.33 (1.33)
6	257,160	28	5.30 (1.33)
7	228,530	32	4.71 (1.18)
8	209,710	36	4.32 (1.08)
9	191,940	40	3.96 (0.99)
10	181,590	44	4.75 (0.94)

^a^ GC integrals were adjusted for pre-existing nitrogen headspace in the reaction vial and partial sampling during the GC measurement.

## Data Availability

Data are available from the authors on request.

## Supplementary Materials

The following supporting information can be downloaded at: https://www.mdpi.com/article/10.3390/nano12050789/s1. Details of instrumentation and procedure, experimental to anchoring synthesis. Video S1: Visible H_2_ formation during irradiation. Scheme S1: Anchoring Ligand Synthesis route. Figures S1–S8: TGA-MS (activated NPs, **1**@TiO_2_), ^1^H NMR spectroscopy (**1**@TiO_2_), solid state IR spectra (activated NPs, **1**@TiO_2_, **1**), solid state absorption spectra (**1**@TiO_2_) and MALDI mass spectra (**1**@TiO_2_). Figures S9–S12: TGA-MS (pristine ZrO_2_, **1**@ZrO_2_), ^1^H NMR spectroscopy (**1**@ZrO_2_), solid state IR spectra (pristine ZrO_2_, **1**@ZrO_2_, Ru@ZrO_2_ and rR@ZrO_2_). Figures S13–S27: TGA-MS, ^1^H NMR spectroscopy, solid state IR spectra, solid state absorption spectra and MALDI mass spectra for Ru@TiO_2_ made with RuCl_3_.3 H_2_O and *cis*-[Ru(bpy)_2_Cl_2_]. Figures S28–S35: TGA-MS, ^1^H NMR spectroscopy, solid state IR spectra, solid state absorption spectra and MALDI mass spectra for Rh@TiO_2_. Figures S36–S49: MALDI mass spectra (rR@TiO_2_, RR@TiO_2_) TGA-MS (rR@TiO_2_, RR@TiO_2_), ^1^H NMR spectroscopy (rR@TiO_2_, RR@TiO_2_), solid state IR spectra (activated NPs, **1**@TiO_2_, rR@TiO_2_, RR@TiO_2_) and solid state absorption spectra (**1**@TiO_2_, Ru@TiO_2_, Rh@TiO_2_, rR@TiO_2_, RR@TiO_2_). Figures S50–S52: TGA-MS (Ru@ZrO_2_ and rR@ZrO_2_) and ^1^H NMR spectroscopy (Ru@ZrO_2_ and rR@ZrO_2_).
