# Supplementary material for: Attraction in Action: Reduction of Water to Dihydrogen Using Surface-Functionalized TiO2 Nanoparticles
Source: Nanomaterials (Basel). 2022 Feb 25;12(5):789. doi: 10.3390/nano12050789 (PMC8912096; doi:10.3390/nano12050789)
Supplement: Supplementary file 1 [file nanomaterials-12-00789-s001.zip › nanomaterials-1584850-supplementary.pdf]

Supplementary Materials

Attraction in Action: Reduction of Water to Dihydrogen Using Surface-Functionalized TiO_2 Nanoparticles

Sven A. Freimann, Catherine E. Housecroft and Edwin C. Constable *

Department of Chemistry, University of Basel, Mattenstrasse 24a, BPR 1096, 4058 Basel, Switzerland; s.freimann@unibas.ch (S.A.F.); catherine.housecroft@unibas.ch (C.E.H.)
* Correspondence: edwin.constable@unibas.ch

1. Experimental Details

1.1 Instruments

^1H NMR spectra were measured at 298 K on a Bruker Avance III-500 NMR spectrometer. ^1H chemical shifts was referenced to residual solvent peaks with respect to δ(TMS) = 0 ppm for ^1H. Reactions and procedures under microwave conditions were carried out in a Biotage Initiator 8 reactor. Microwave vials (5 mL and 20 mL) were from Biotage and were selected depending on the required solvent volume. For centrifugation an Eppendorf Centrifuge 5415 R was used with 2-, 10- and 50 mL samples. Irradiation experiments were done in a Biotage 5 mL microwave vial with a LOT Quantum Design LS0811 instrument as light source at 1200 W m^{-2}. The simulated light power was calibrated with a silicon reference cell. Solid-state absorption spectra were recorded on an Agilent Cary 5000 spectrophotometer with a Diffuse Reflectance Accessory. For each solid-state absorption spectrum, a baseline correction was done with activated NPs as the reference sample. FTIR spectra were recorded on a Perkin Elmer UATR Two spectrophotometer. MALDI mass spectra were measured using a Shimadzu MALDI-8020 with α-cyano-4-hydroxycinnamic acid (CHCA) solution as matrix for sample preparation. Thermogravimetric analysis (TGA) was performed on a TGA5500 (TA Instruments) instrument under nitrogen and coupled to a MKII mass spectrometer. Initially, the temperature was held at 30 °C for 10 min before heating at a rate of 10 °C/min to 120 °C. This temperature was maintained for 30 min to remove all traces of water. Afterwards the sample was heated to 900 °C at a rate of 10 °C/min. After maintaining the temperature at 900 °C for 30 min, the sample was cooled to ambient temperature. Autoclave reactions were done in a 23 mL Acid digestion vessel (Parr Instrument Company) using a forced convection chamber furnace (Nabertherm NA15/65).

Gas chromatography was performed on a Model 8610C Gas Chromatograph from SRI instruments equipped with a flame ionization detector (FID/meth 300C hi gain), thermal conductivity detector (TCD100C low current), Haysep D 3 m 1/8′′ AD 2 mm ID Mesh 80/100 column and a HT2000H headspace autosampler from HTA S.R.L. The method used a constant column temperature of 90°C, 3 minutes event time, N_2 carrier gas at 10 mL min^{-1} and 1 mL isocratic sampling. The GC integral was measured, adjusted for pre-existing nitrogen volume, sampling amount and then converted to volume in mL using a H_2 GC measured blank curve. Fluorescence emission spectra were measured on a Shimadzu RF-6000 spectrofluorometer with an excitation slit width of 3 – 10 nm and an emission slit width of 3 – 10 nm. For the emission spectroscopy, the NPs were dispersed in water with a concentration of 0.1 mg / mL. NP sample analysis with triple quadrupole inductively coupled plasma mass spectrometry (ICP-QQQ-MS) was performed on an 8800 ICP-QQQ MS system (Agilent) using the general-purpose operational settings. Samples were digested in aqueous HNO_3 (69%, semiconductor grade, PanReac AppliChem), diluted with milliQ water to a final HNO_3 concentration of 3 % and quantification was performed via multi-element standards (Sigma-Aldrich). To account for matrix effects ^{89}Y was used as an internal standard. ^{47}Ti, ^{101}Ru and ^{103}Rh were quantified operating the ICP-MS system in single-quad mode using helium as the collision gas. Commercial ZrO_2 NPs (Sigma-Aldrich, <100 nm TEM) were further characterized using TGA-MS and

FTIR spectroscopy. TGA: weight loss / %, 0.8 (200–900°C). TGA-MS: amu, 18 (200–900 °C). FTIR spectroscopy: 1629, 766, 683, 582 and 467 cm^{-1}.

1.2 Anchoring Ligand Synthesis

The anchoring ligand (**1**) and its precursors were prepared according to the literature (see Scheme S1 and following sections). The respective spectroscopic data matched those previously reported.

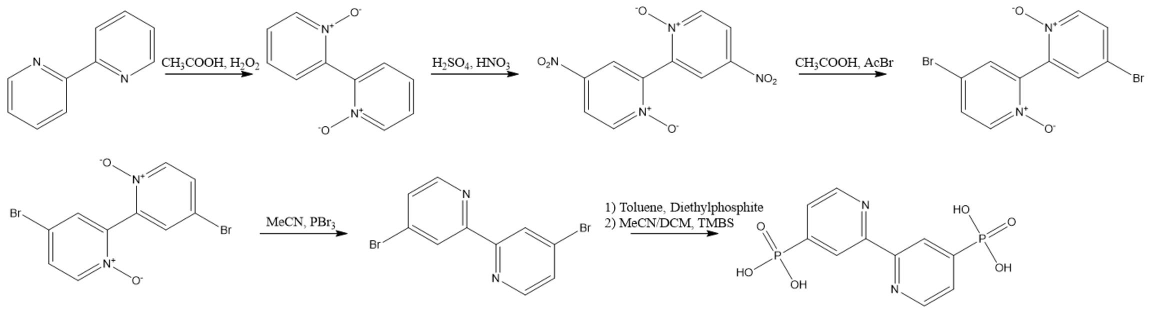

Scheme S1. Anchoring ligand synthesis route.

2,2′-Bipyridine *N,N′*-dioxide[1]

H_2O_2 (35%, 35 mL) was added to 2,2′-bipyridine (12.0 g, 76.8 mmol) in glacial acetic acid (80 mL) and then stirred at 75°C for 48 h. After cooling to room temperature acetone (1 L) was added and the suspension was cooled to 4°C for 3h. The precipitate was filtered off, washed with acetone (5 x 25 mL) and then tried at high vacuum. 2,2′-bipyridine *N,N′* dioxide (13.4 g , 70.9 mmol , 92%) was isolated as white solid. ^1H NMR (600 MHz, D_2O, 298 K) δ /ppm: 8.45(m, 2H), 7.83 (m, 2H), 7.74 (m, 2H).

4,4′-Dinitro-2.2′-bipyridine *N,N′*-dioxide[1]

2,2′-Bipyridine *N,N′* dioxide (13.4 g , 70.9 mmol) dissolved in fuming sulphuric acid (39 mL). The solution was cooled to 0°C and then fuming nitric acid (30 mL) was slowly added. The mixture was then stirred at 100°C for 7h and then slowly purred on ice (300 g). The precipitate was filtered off, washed with water (5 x 100 mL) and then tried at high vacuum. The product 4,4′-dinitro-2.2′-bipyridine *N,N′* dioxide (16.4 g, 59.0 mmol, 83%) was isolated as beige powder. ^1H NMR (600 MHz, DMSO-d_6, 298 K) δ /ppm: 8.70 (d, *J* = 3.3, 2H), 8.60 (d, *J* = 7.2, 2H), 8.38 (dd, *J* = 7.2, 3.3, 2H).

4,4'-Dibromo-2,2'-bipyridine *N,N′*-dioxide[2]

4,4′-Dinitro-2.2′-bipyridine *N,N′* dioxide (8.40 g, 30.2 mmol) was added to glacial acetic acid (120 mL) and stirred at 60°C. Acetyl bromide (65.5 mL, 884 mmol) was added and the solution was stirred at reflux for 2h. The solution was cooled to r.t. and then poured on ice (500 g). After neutralisation with NaOH the precipitate was filtered off and washed with water exhaustively. The crude beige product (8.19 g) was used for the next step without further purification.

4,4'-Dibromo-2,2'-bipyridine[3]

4,4'-Dibromo-2,2'-bipyridine *N,N′* dioxide (8.00 g, 23.1 mmol) was added to anhydrous acetonitrile (100 mL) and cooled down to 0°C. PBr_3 (32 mL, 341 mmol) was added and the solution was refluxed while stirring for 4h. After cooling down the reaction was carefully poured on ice (200 g) and then NaOH was added till pH 11 was reached. The suspension was extracted with $CHCl_3$ (5 x 100 mL), dried with $MgSO_4$, the solvent was then evaporated under reduced pressure and the solid was dried at high vacuum. 4,4'-dibromo-2,2'-bipyridine (6.58 g, 20.9 mmol, 91%) was isolated as off white powder. ^1H NMR (500 MHz, $CDCl_3$, 298 K) δ /ppm: 8.62 (dd, *J* = 2.0, 0.6, 2H), 8.49 (d, *J* = 5.2, 0.6 2H), 7.51 (dd, *J* = 5.2, 2.0, 2H).

Tetraethyl [2,2′-bipyridine]-4.4′-diylbis(phosphonate)[4]

4,4'-Dibromo-2,2'-bipyridine (4.01 g, 12.7 mmol) and tetrakis(tiphenylphosphine) palladium(0) (1.37 g, 1.19 mmol) were added to dry under flask under nitrogen. The solids were dissolved with anhydrous toluene (40 mL). Diethylphosphine (4.68 mL, 36.3 mmol) and triethylamine (5.19 mL, 37.3 mmol) were added to the reaction under stirring with a syringe. The solution was stirred at 110°C for 3.5 h, filtered hot to remove trimethylamine hydrobromide and then filtered again after the reaction cooled down. The solvent was removed under reduced pressure and the residue was recrystallized from hexanes. Tetraethyl [2,2′-bipyridine]-4.4′-diylbis(phosphonate) (4.56g, 10.7 mmol, 83%) was isolated as white crystals. ^1H NMR

(500 MHz, $CDCl_3$, 298 K) δ /ppm: 8.84 (td, *J* = 4.8, 1.4, 2H), 8.78 (dd, *J* = 13.5, 1.4 2H), 7.73 (ddd, *J* = 13.5, 4.8, 1.4, 2H), 4.19 (m, 8H), 1.36 (t, *J* = 7.1, 12H).

[2,2′-Bipyridine]-4.4′-diylbis(phosphonic acid), (1)[5]

Tetraethyl [2,2′-bipyridine]-4.4′-diylbis(phosphonate) (1.50 g, 3.50 mmol) was dissolved in dry acetonitrile, dichloromethane (50 mL, 1:1) under nitrogen. Bromotrimethylsilane (6 mL, 45.5 mmol) was added and the solution was refluxed under nitrogen overnight. A pink precipitate formed and the reaction mixture was cooled to room temperature. Water was added dropwise to the reaction mixture while stirring, the precipitate was filtered off and washed with cold MeOH (3 x 2 mL) and cold Ether (3 x 10 mL). [2,2′-Bipyridine]-4.4′-diylbis(phosphonic acid) was isolated as off white powder (0.95 g, 3.00 mmol, 86%). ^1H NMR (500 MHz, D_2O, 298 K) δ /ppm: 8.92 (m, 2H), 8.68 (d, *J* = 12.3, 2H), 8.12 (m, 2H).

1.3 Supporting Figures

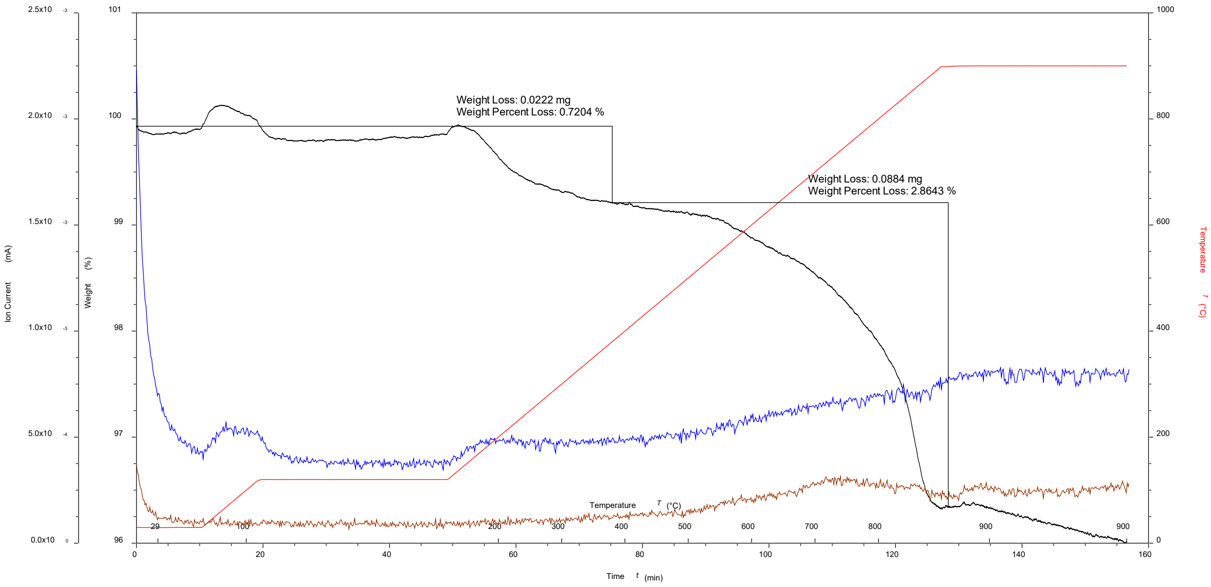

Figure S1. TGA-MS curves for **1**@TiO_2: Weight against time and temperature (black), temperature (red), amu 18 (blue), amu 44 (brown).

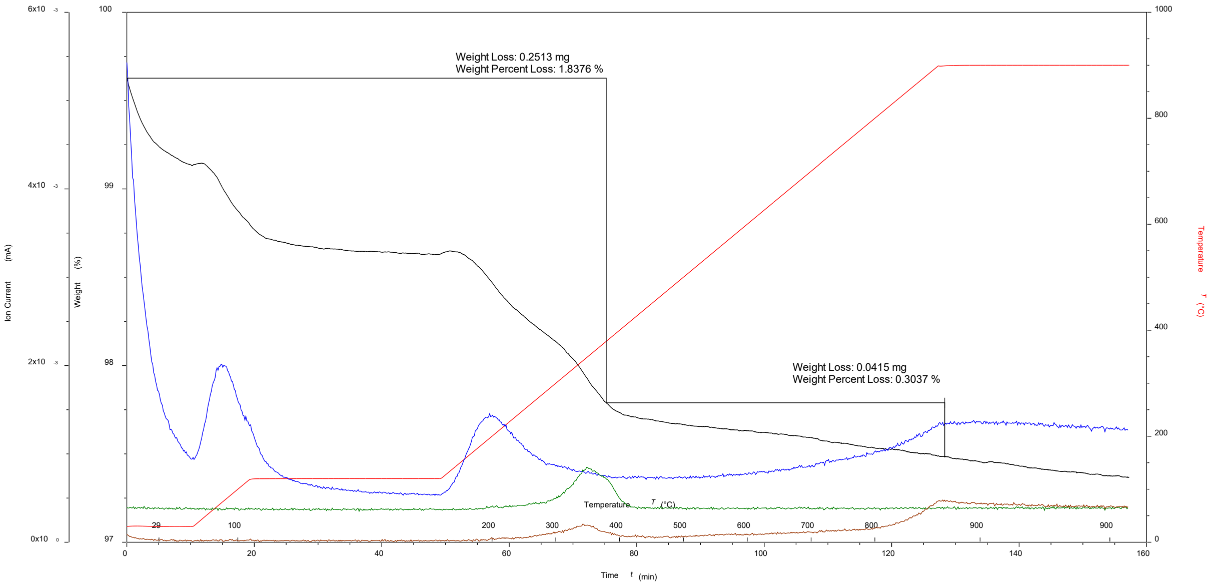

Figure S2. TGA-MS curves for a-NPs: Weight against time and temperature (black), temperature (red), amu 18 (blue), amu 30 (green), amu 44 (brown). Adapted figure from previous work.[6]

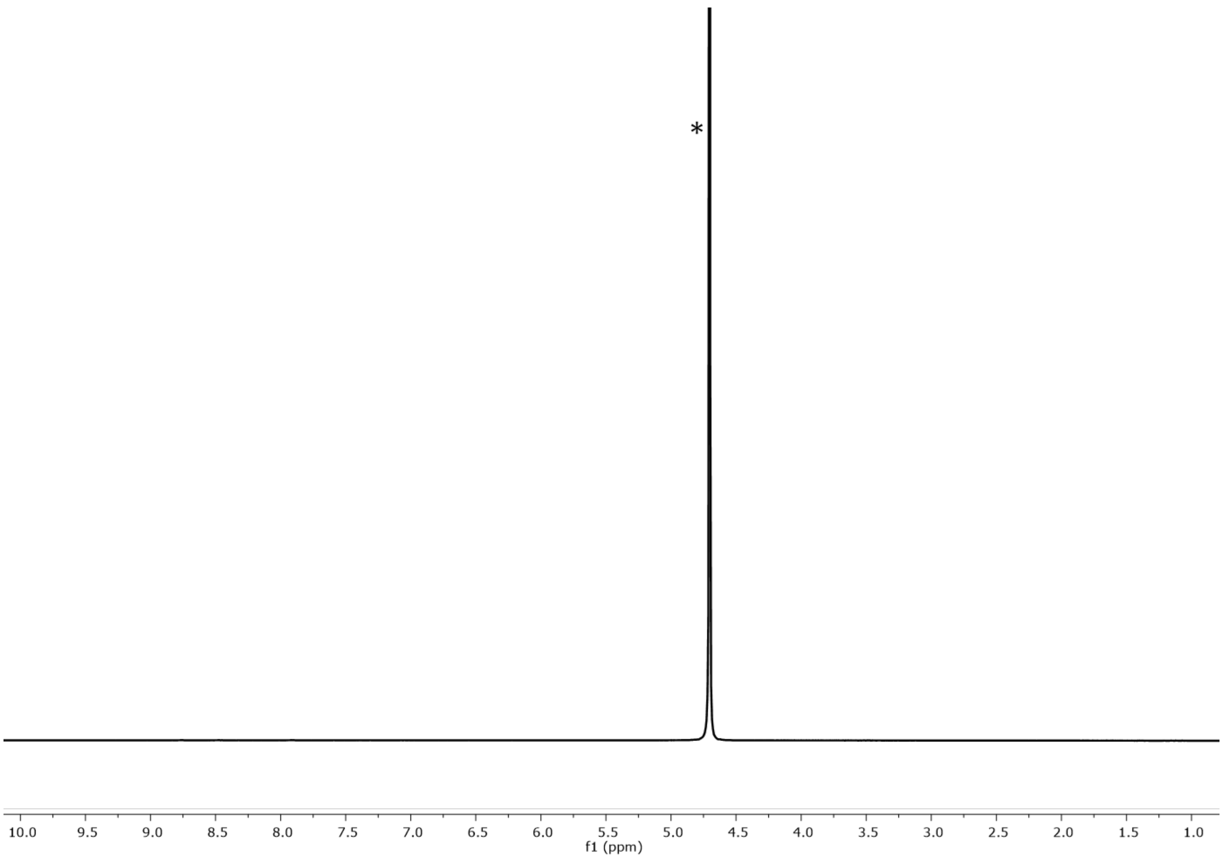

Figure S3. ^{1}H NMR (500 MHz, D_2O, 298 K) spectrum of **1**@TiO_2, * = HOD. Chemical shifts in δ /ppm.

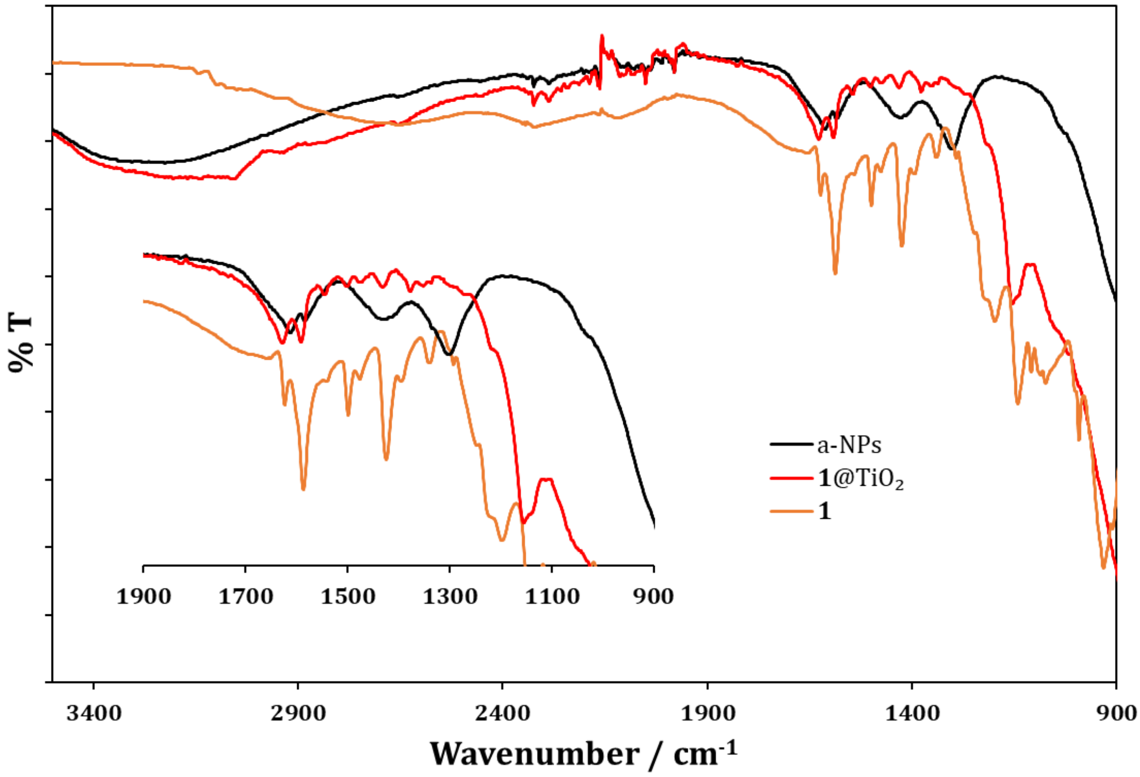

Figure S4. Solid-state IR spectra of **1**@TiO_2 (red), a-NPs (black) and pristine anchoring ligand **1** (orange) with expansion of the low energy regions.

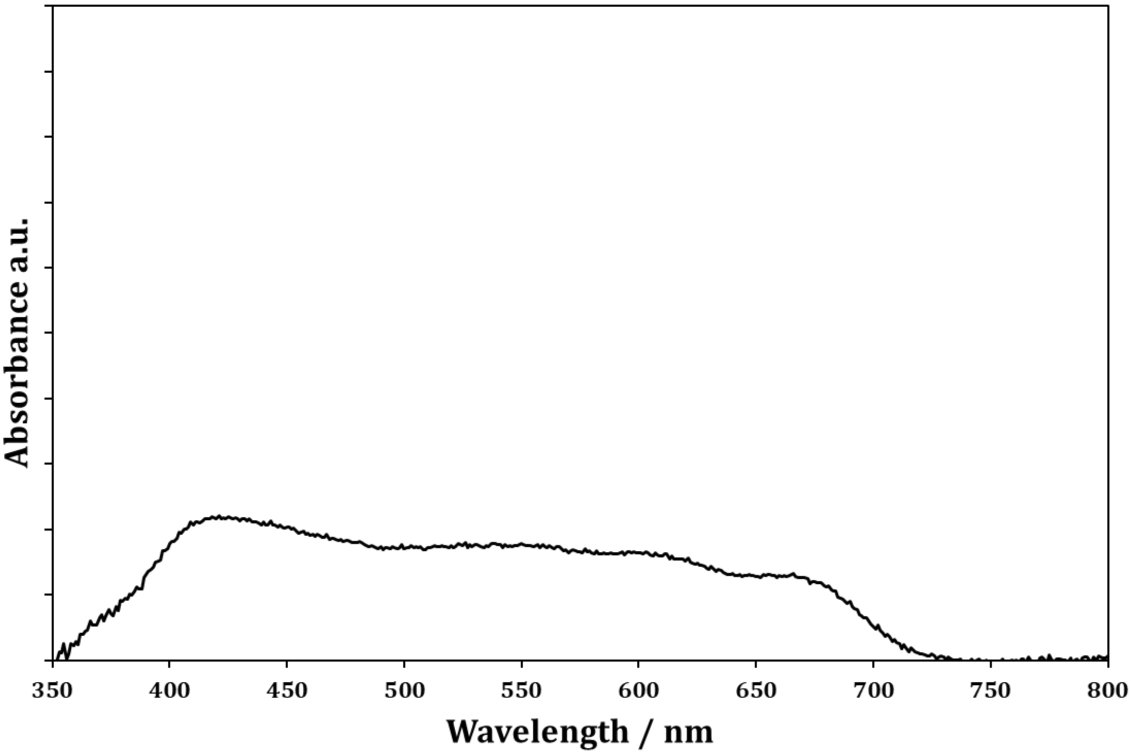

Figure S5. Solid-state absorption spectra of **1**@TiO_2 using a-NP as reference.

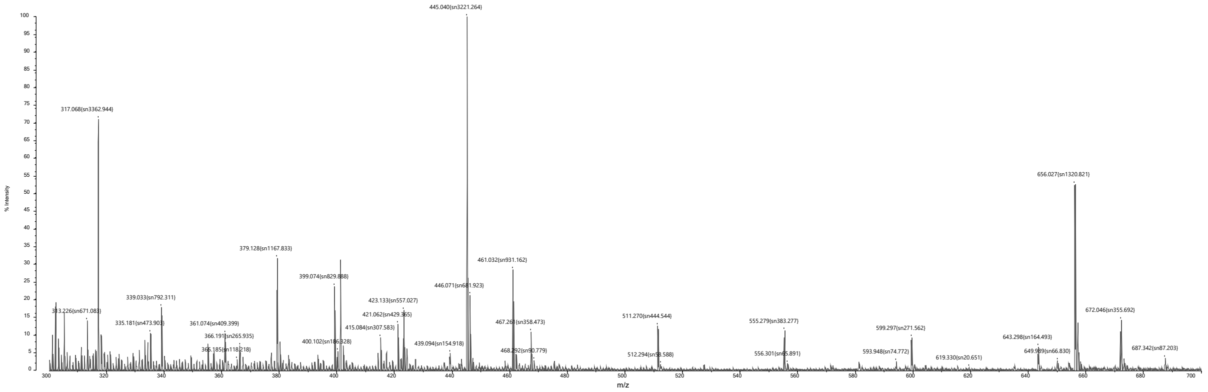

Figure S6. MALDI mass spectrum (with CHCA matrix) of **1**@TiO_2.

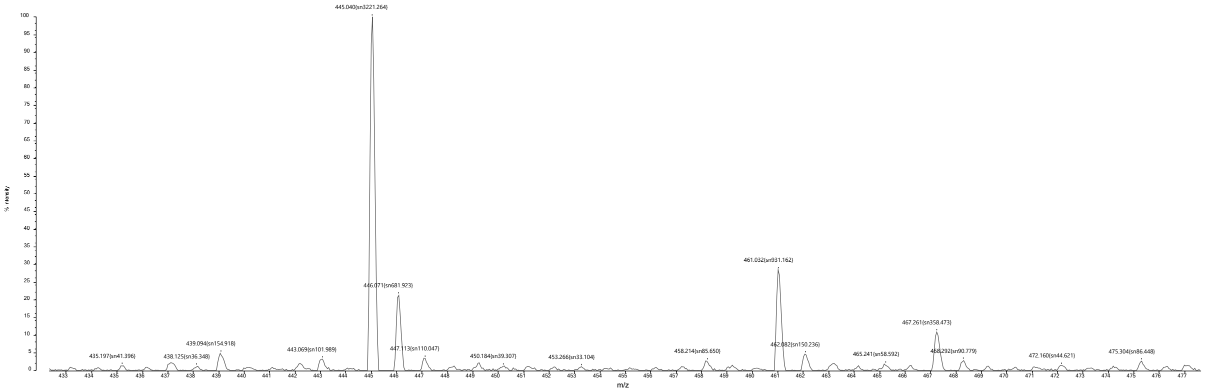

Figure S7. MALDI mass spectrum (with CHCA matrix) of **1**@TiO_2.

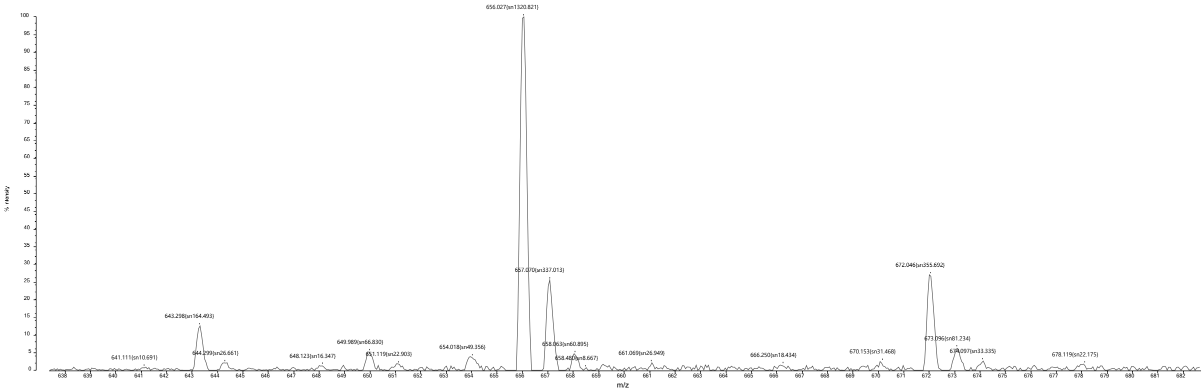

Figure S8. MALDI mass spectrum (with CHCA matrix) of **1**@TiO_2.

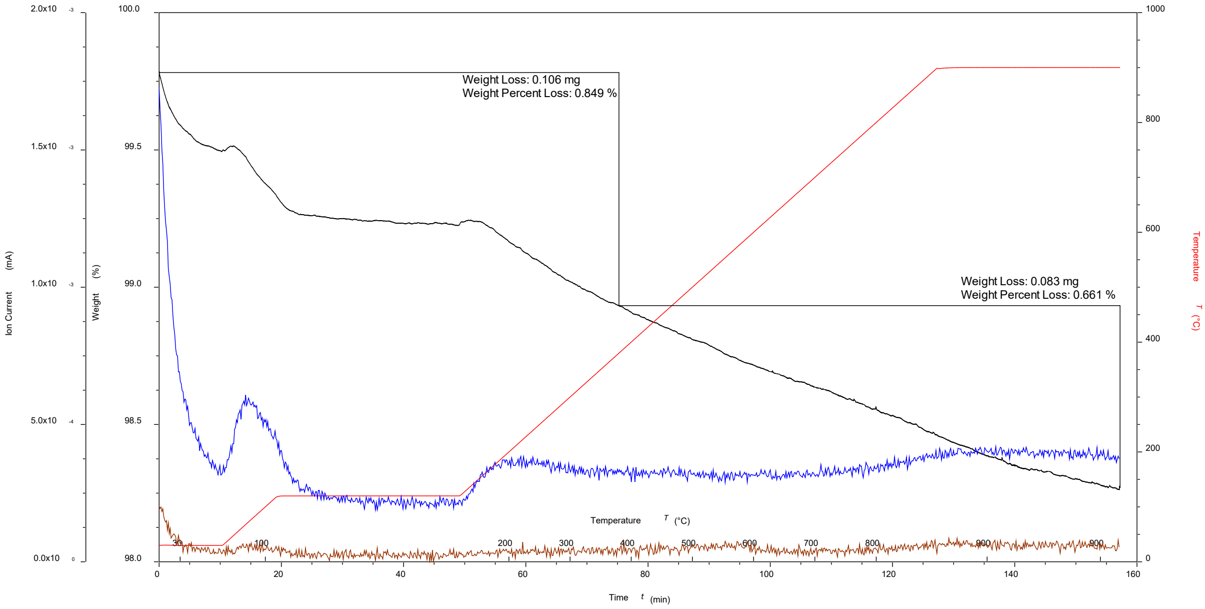

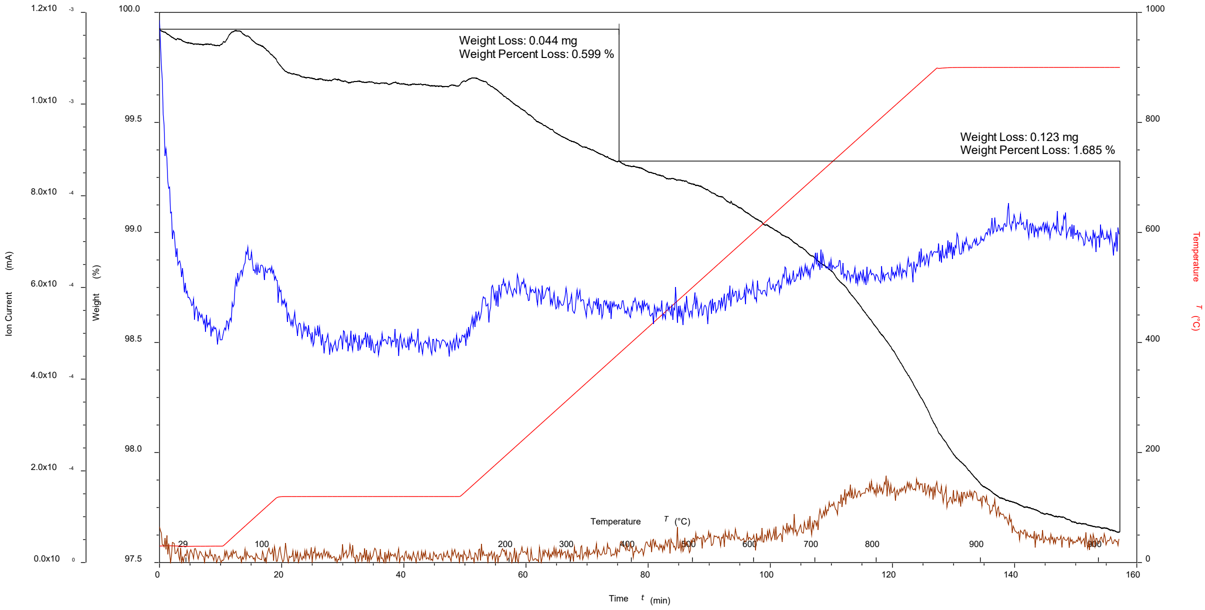

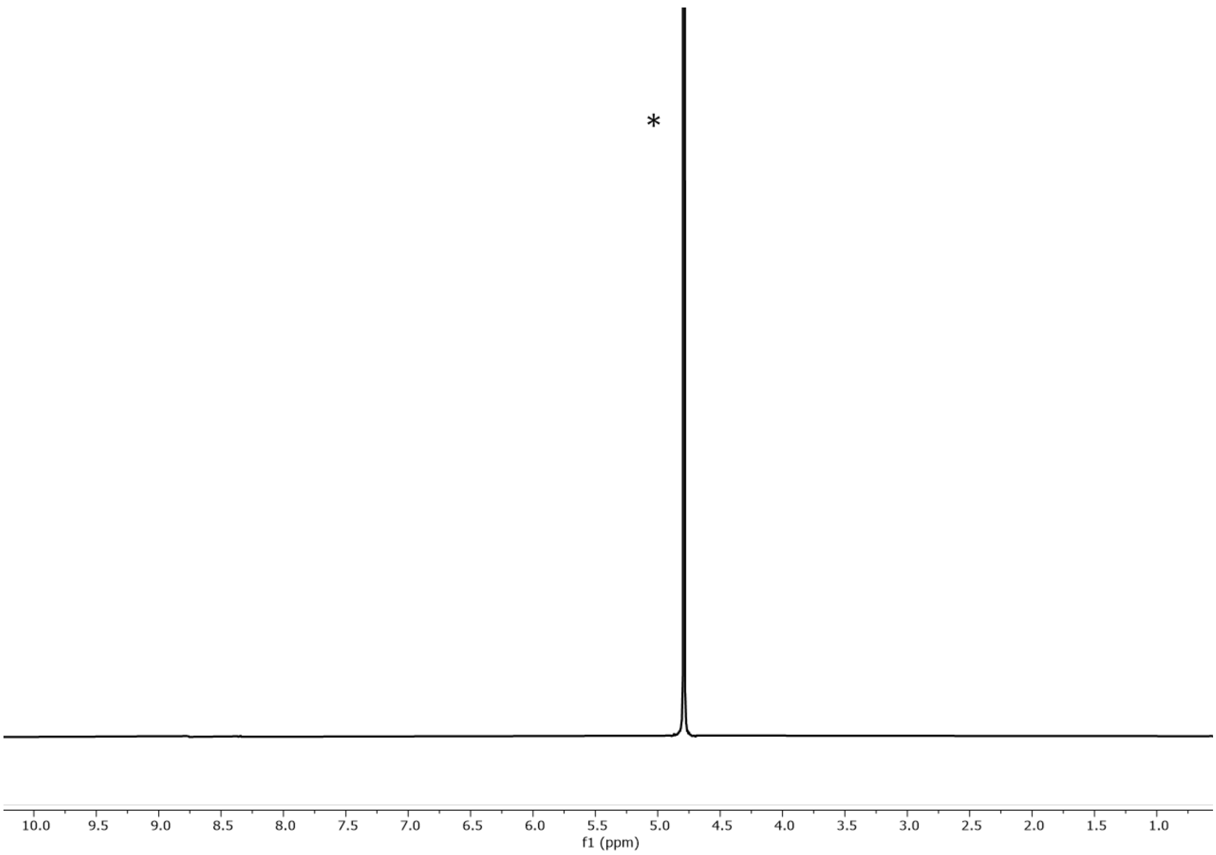

Figure S11. ^{1}H NMR (500 MHz, D_2O, 298 K) spectrum of **1**@ZrO_2, * = HOD. Chemical shifts in δ /ppm.

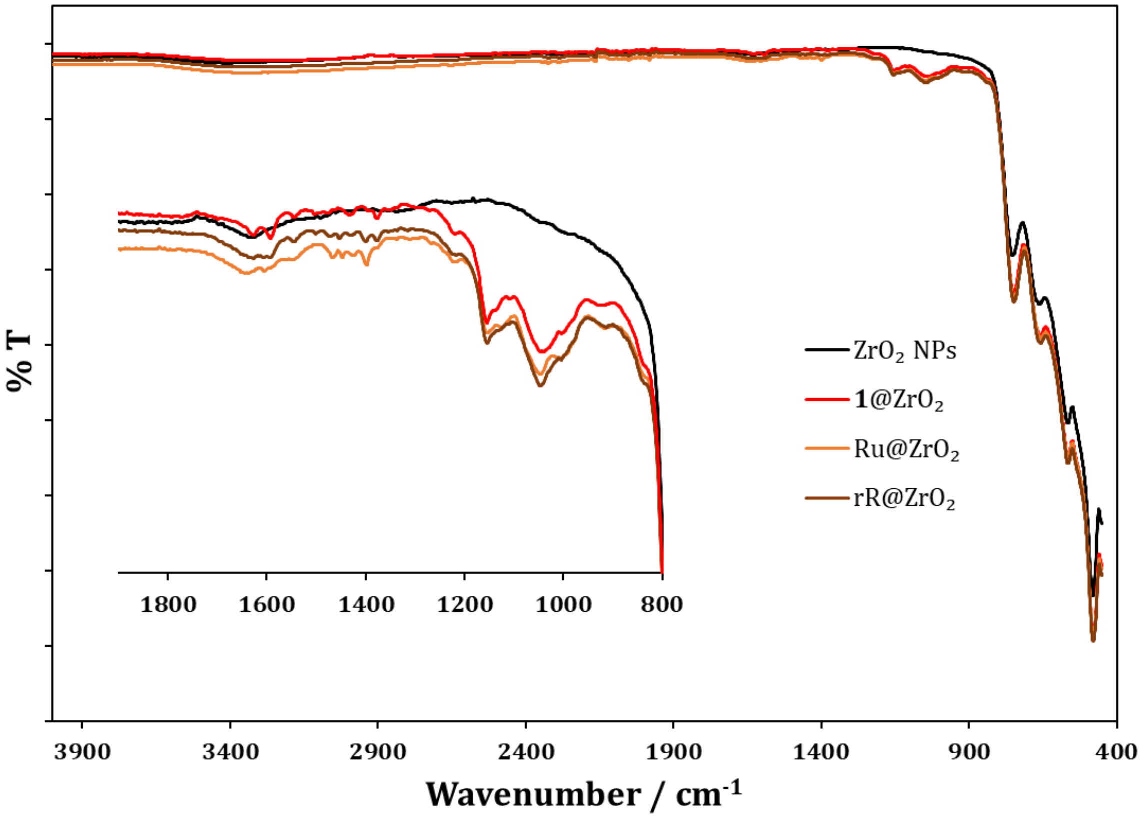

Figure S12. Solid-state IR spectra of pristine ZrO_2 NPs (black), **1**@ZrO_2 (red), Ru@ZrO_2 (orange), rR@ZrO_2 (brown) with expansion of the low energy regions.

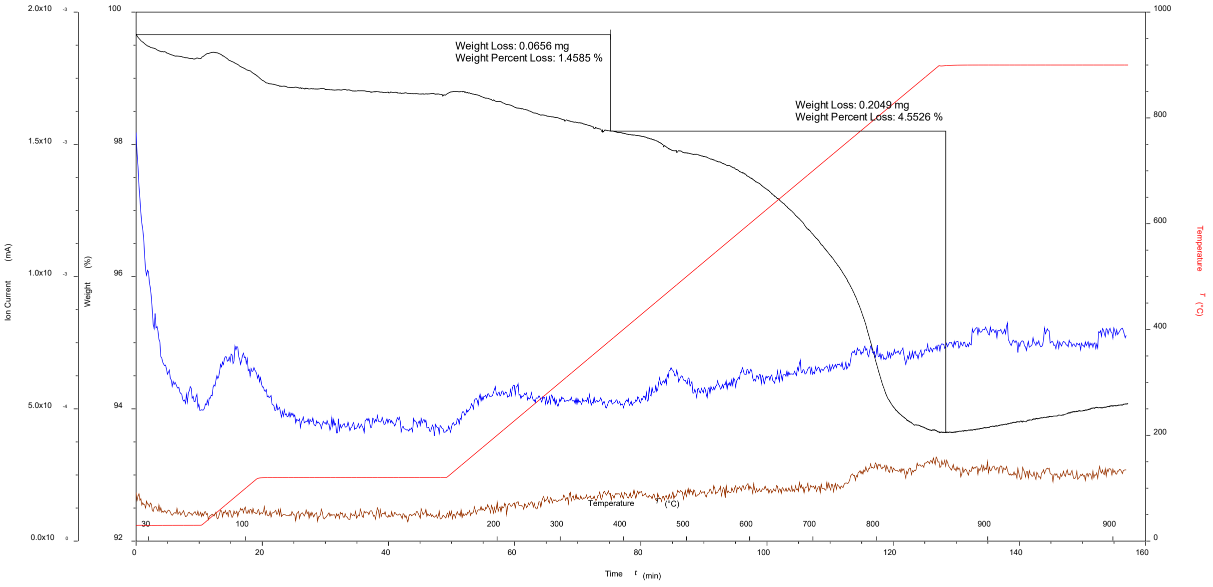

Figure S13. TGA curves for Ru@TiO2 made with $RuCl_3{\cdot}3H_2O$ and bpy: Weight against time and temperature (black), temperature (red), amu 18 (blue), amu 44 (brown).

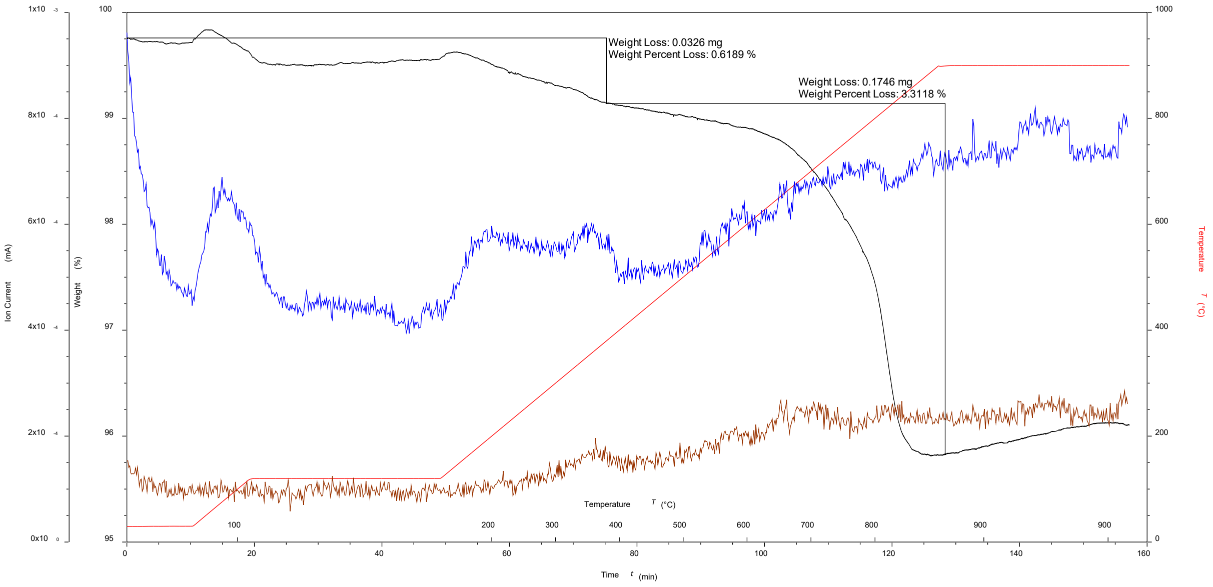

Figure S14. TGA curves for Ru@TiO2 made with *cis*-[Ru(bpy)$_2$Cl$_2$]: Weight against time and temperature (black), temperature (red), amu 18 (blue), amu 44 (brown).

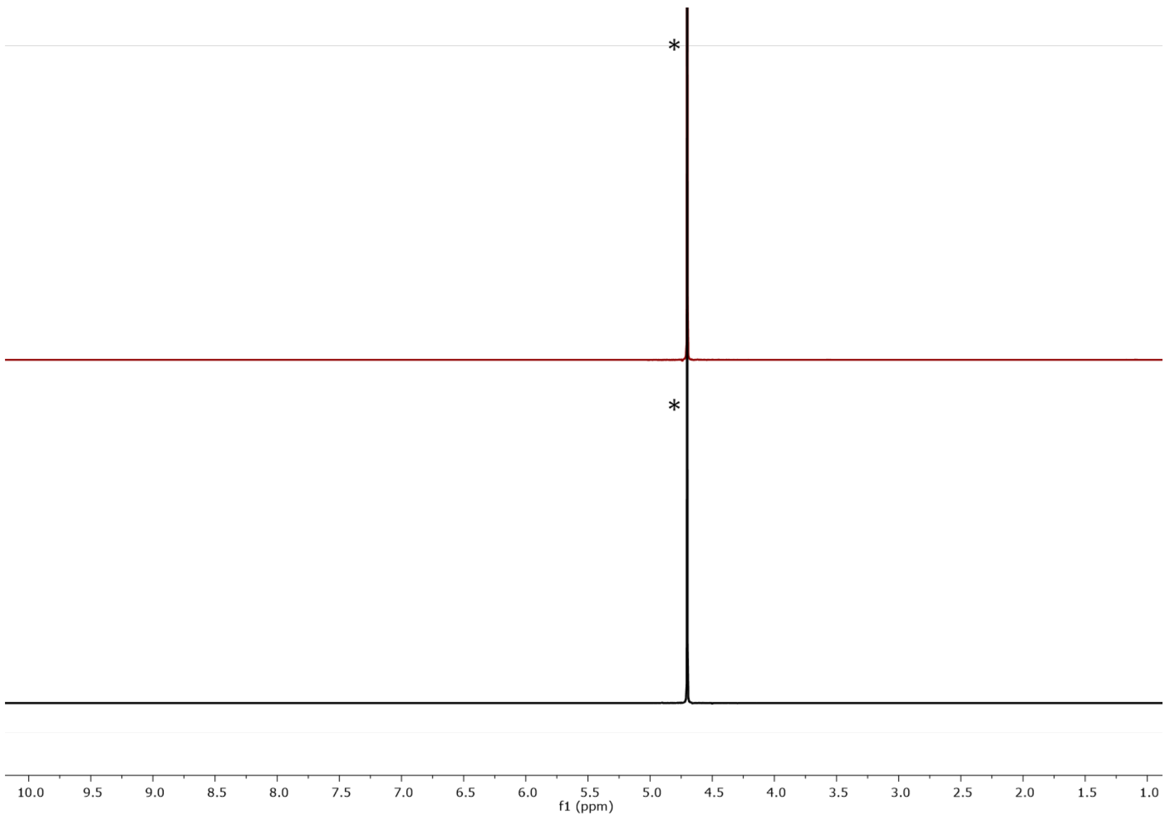

Figure S15. ^1H NMR (500 MHz, D_2O, 298 K) spectrum of Ru@TiO_2 made with: $RuCl_3{\cdot}3H_2O$ and bpy (black), *cis*-[Ru(bpy)$_2$Cl$_2$] (red):, * = HOD. Chemical shifts in δ /ppm.

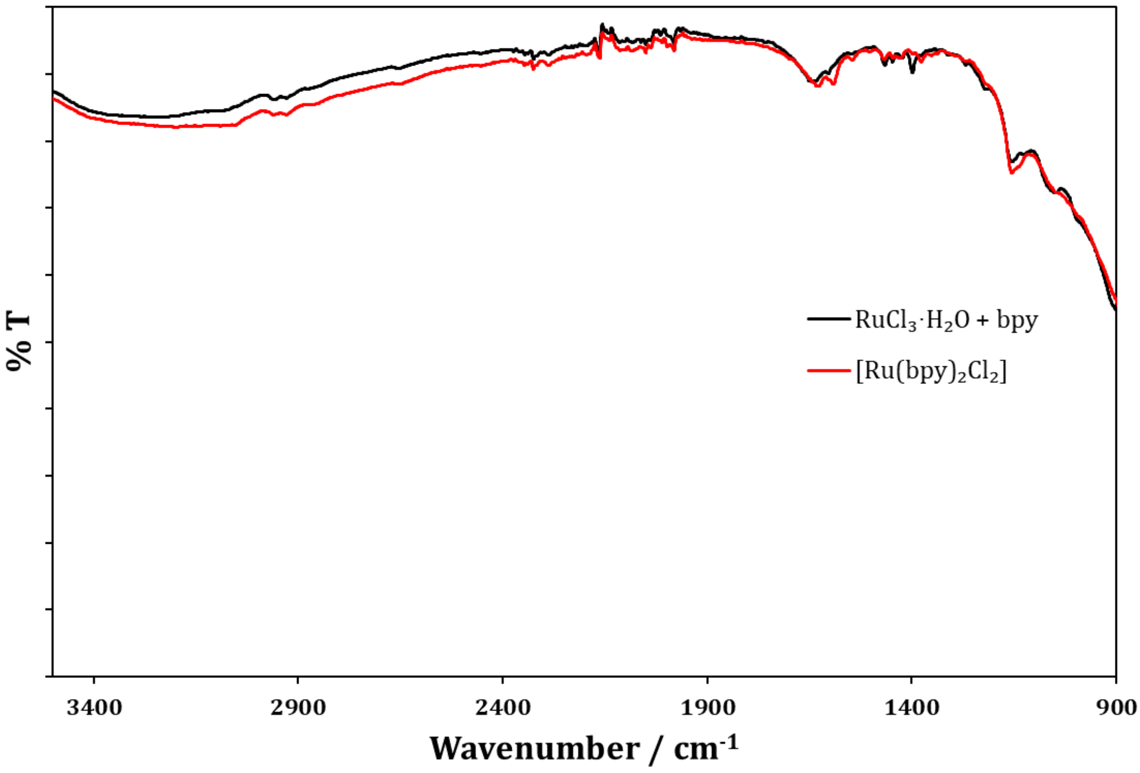

Figure S16. Solid-state IR spectra of Ru@TiO_2 made with: $RuCl_3{\cdot}3H_2O$ and bpy (black), *cis*-[Ru(bpy)$_2$Cl$_2$] (red).

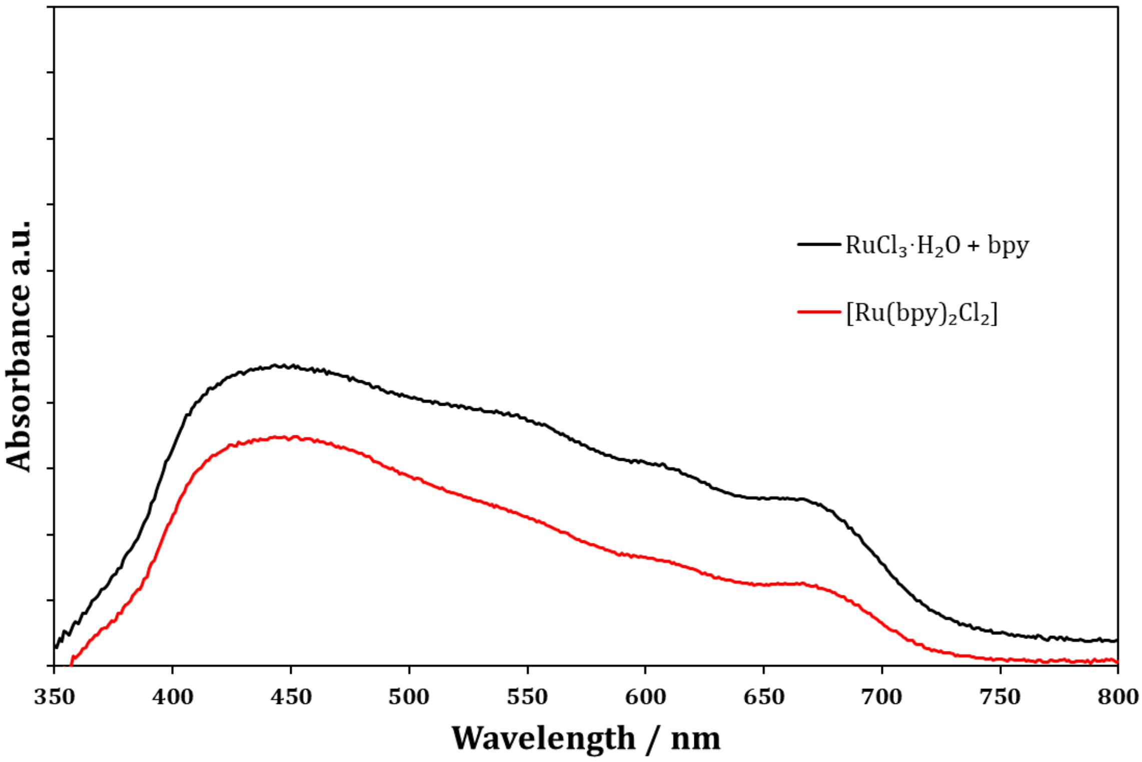

Figure S17. Solid-state absorption spectra of Ru@TiO2 made with: $RuCl_3{\cdot}3H_2O$ and bpy (black), *cis*-$[Ru(bpy)_2Cl_2]$ (red) using a-NP as reference.

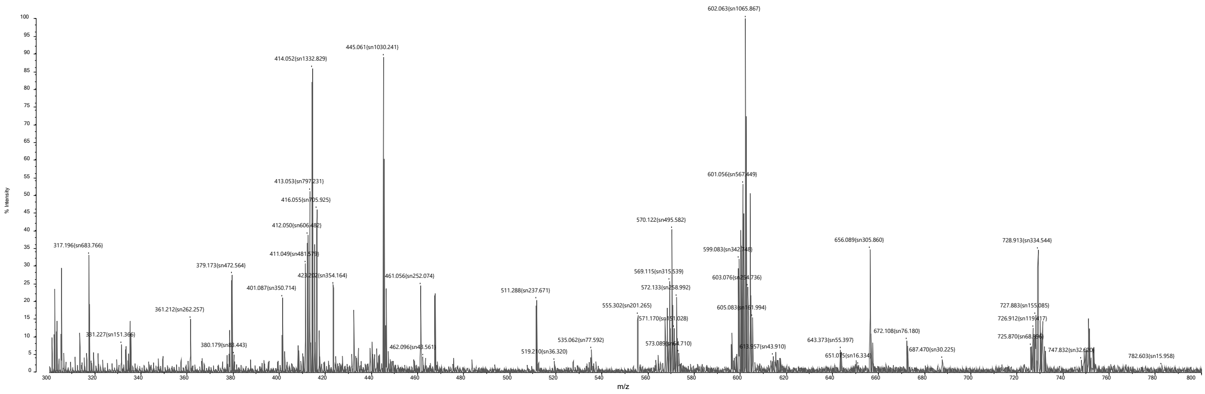

Figure S18. MALDI mass spectrum (with CHCA matrix) of Ru@TiO2 with $RuCl_3{\cdot}3H_2O$.

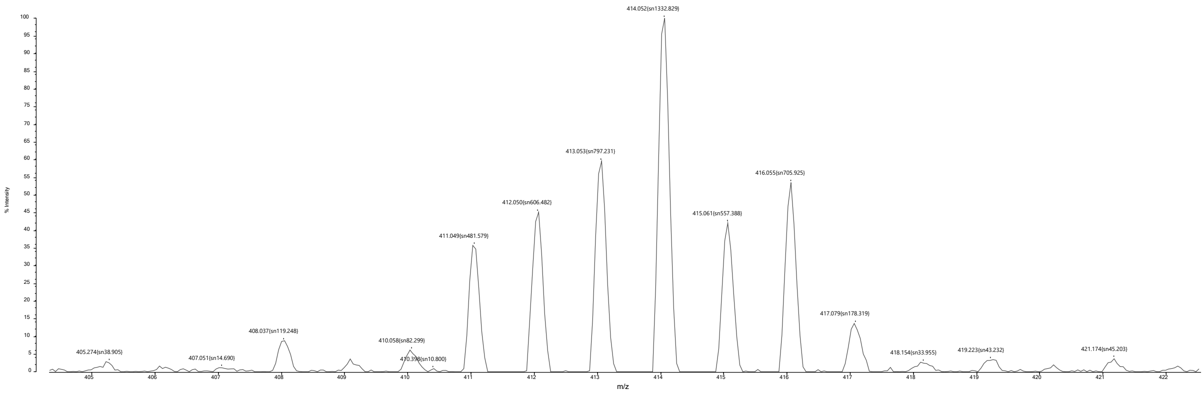

Figure S19. MALDI mass spectrum (with CHCA matrix) of Ru@TiO2 with $RuCl_3{\cdot}3H_2O$.

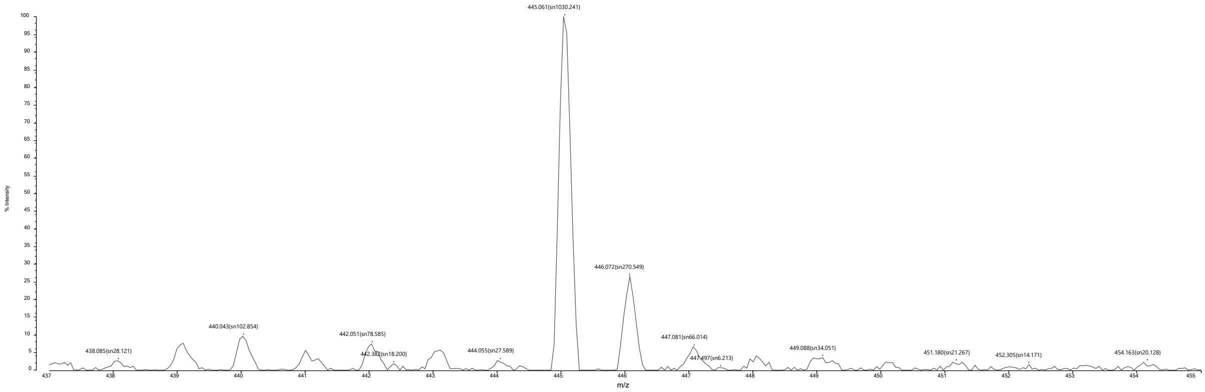

Figure S20. MALDI mass spectrum (with CHCA matrix) of Ru@TiO2 with RuCl3·3H2O.

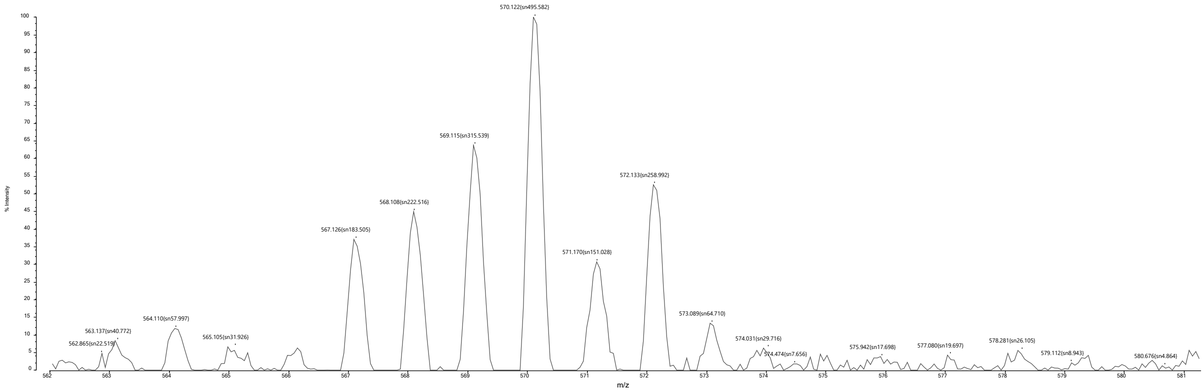

Figure S21. MALDI mass spectrum (with CHCA matrix) of Ru@TiO2 with RuCl3·3H2O.

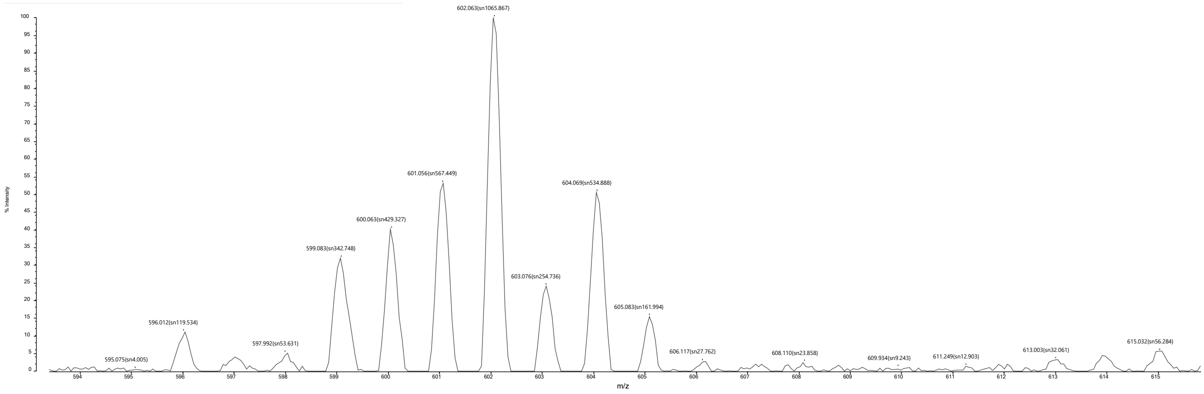

Figure S22. MALDI mass spectrum (with CHCA matrix) of Ru@TiO2 with RuCl3·3H2O.

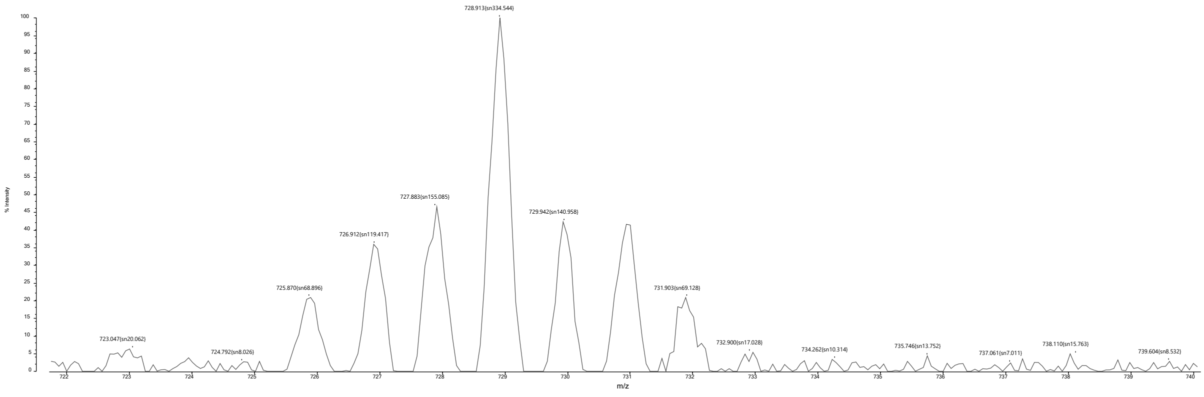

Figure S23. MALDI mass spectrum (with CHCA matrix) of Ru@TiO2 with RuCl3·3H2O.

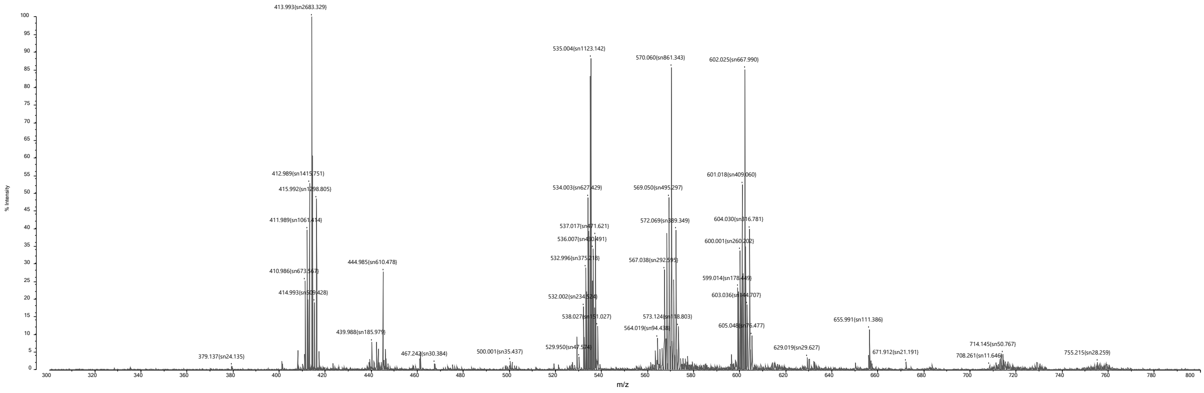

Figure S24. MALDI mass spectrum (with CHCA matrix) of Ru@TiO_2 with *cis*-[Ru(bpy)$_2$Cl$_2$].

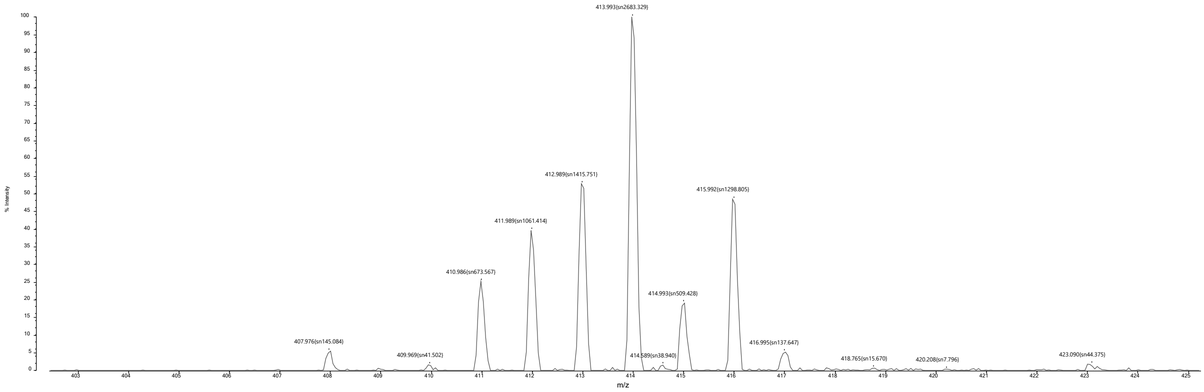

Figure S25. MALDI mass spectrum (with CHCA matrix) of Ru@TiO_2 with *cis*-[Ru(bpy)$_2$Cl$_2$].

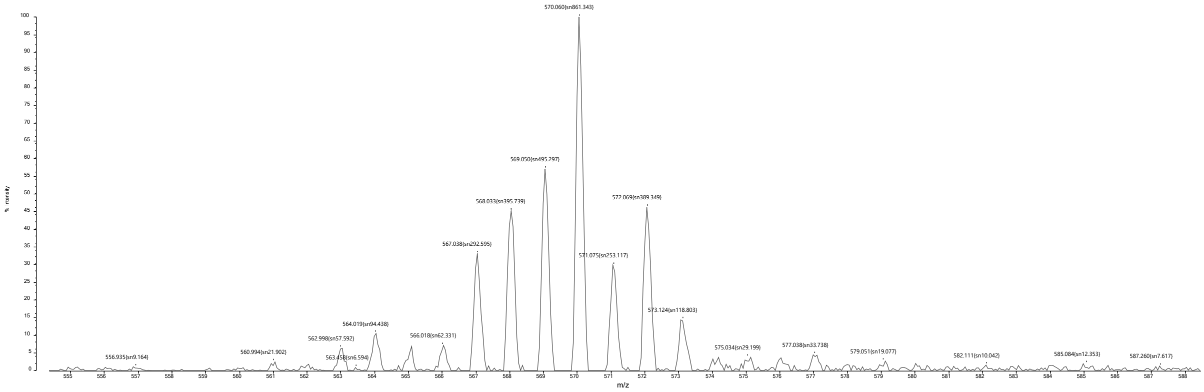

Figure S26. MALDI mass spectrum (with CHCA matrix) of Ru@TiO_2 with *cis*-[Ru(bpy)$_2$Cl$_2$].

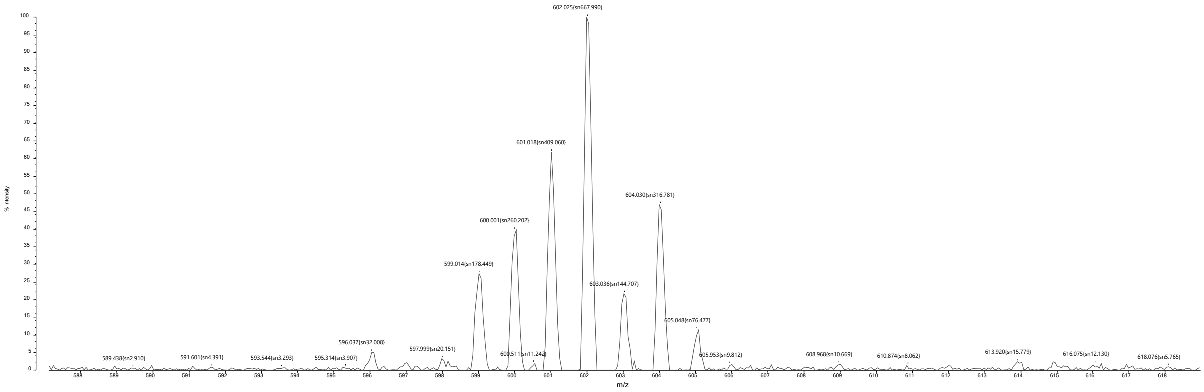

Figure S27. MALDI mass spectrum (with CHCA matrix) of Ru@TiO_2 with *cis*-[Ru(bpy)$_2$Cl$_2$].

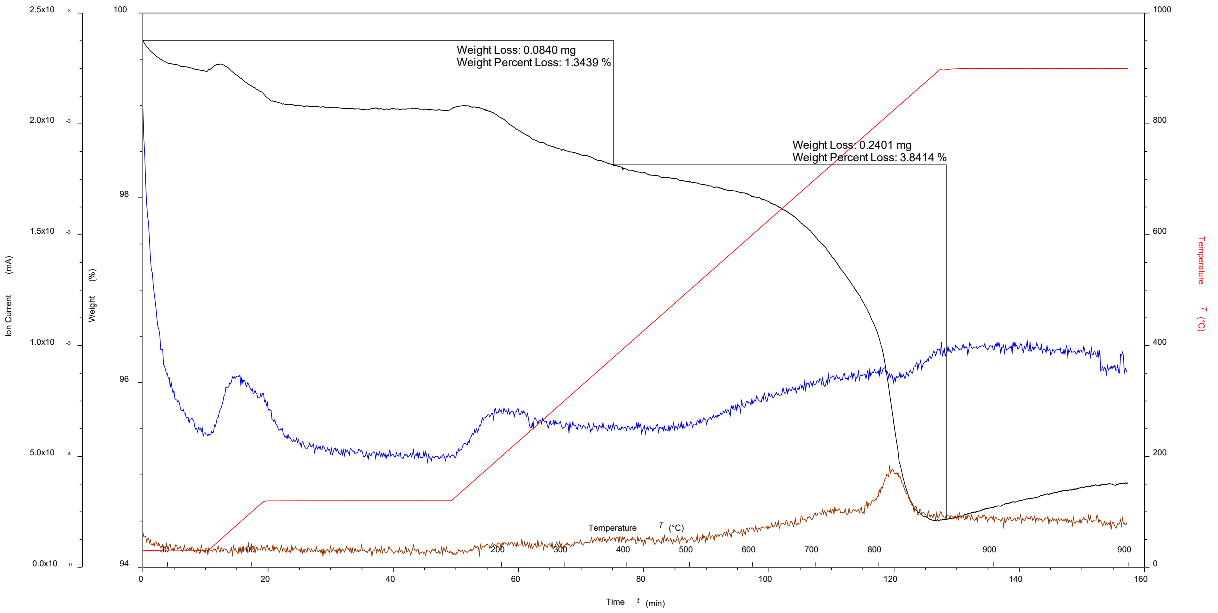

Figure S28. TGA curves for Rh@TiO2: Weight against time and temperature (blue), derivative of weight change against time (red).

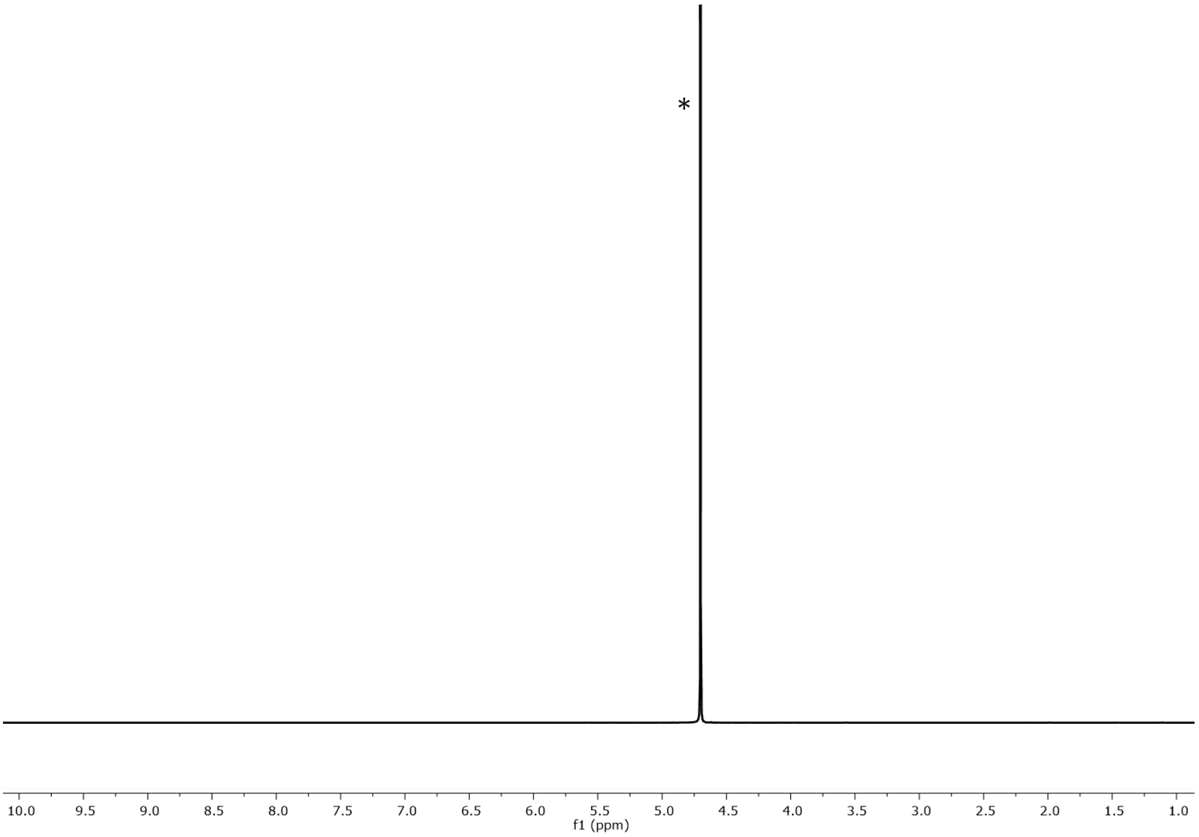

Figure S29. ^{1}H NMR (500 MHz, D_2O, 298 K) spectrum of Rh@TiO_2, * = HOD. Chemical shifts in δ /ppm.

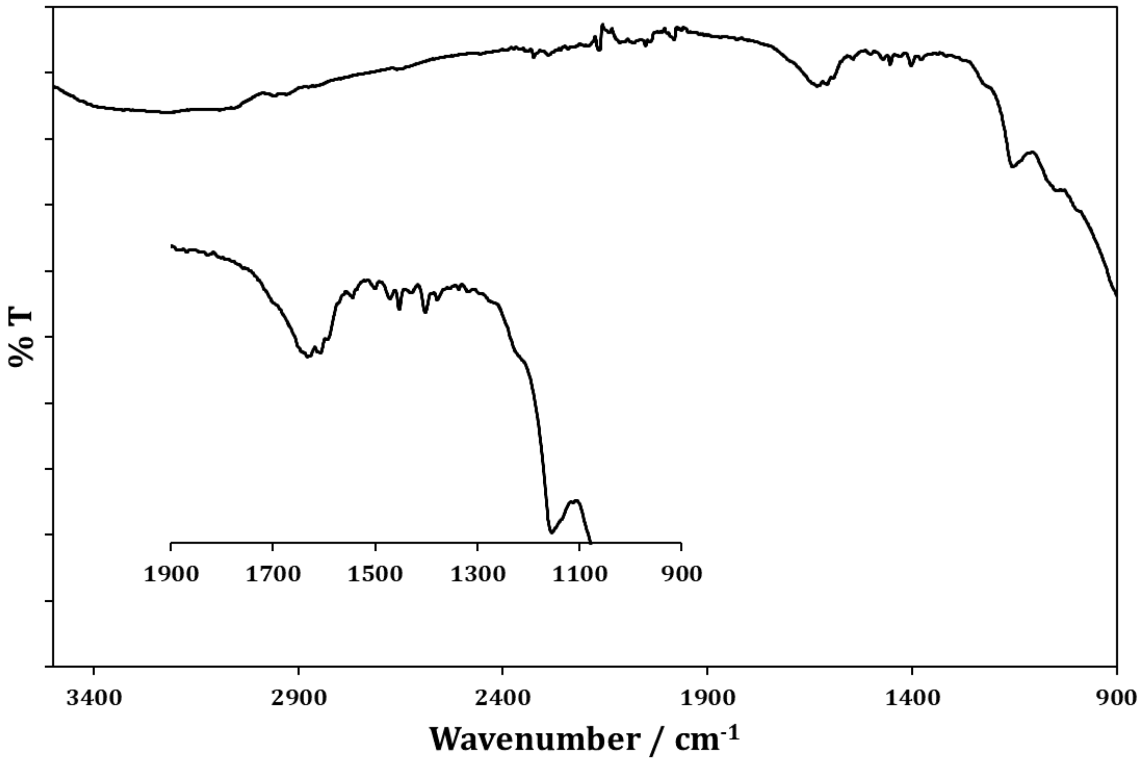

Figure S30. Solid-state IR spectrum of Rh@TiO_2 with expansion of the low energy regions.

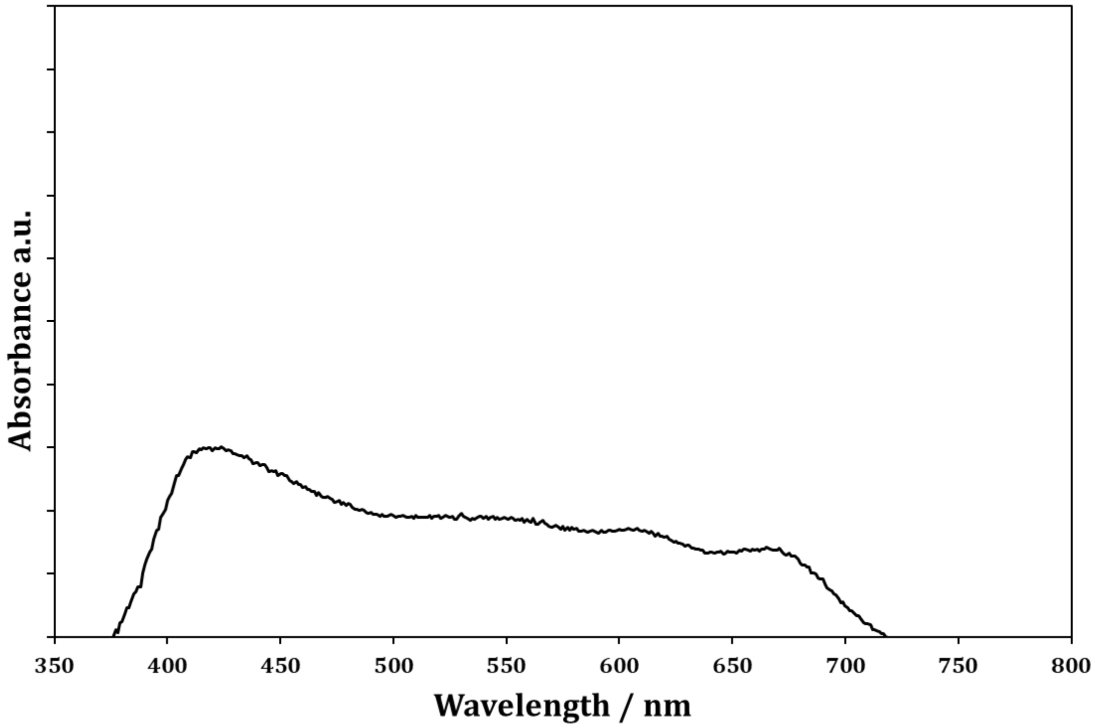

Figure S31. Solid-state absorption spectra of Rh@TiO_2 using a-NP as reference.

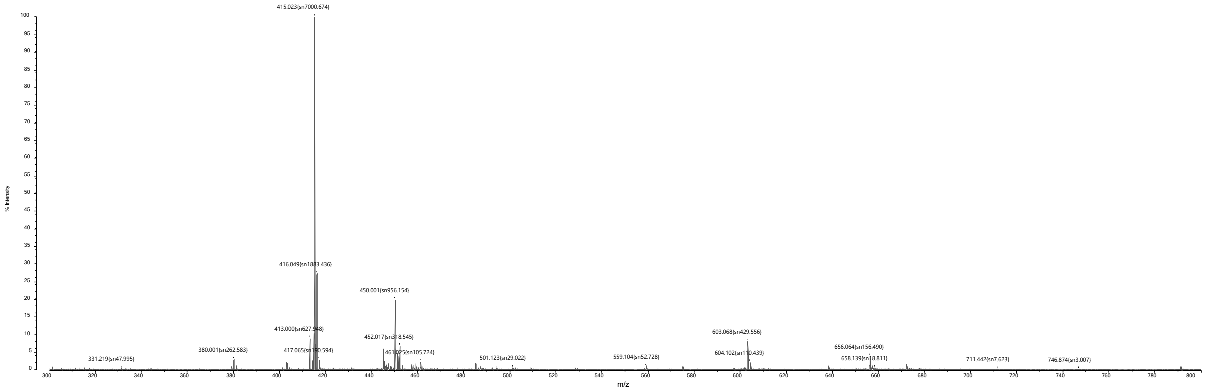

Figure S32. MALDI mass spectrum (with CHCA matrix) of Rh@TiO_2.

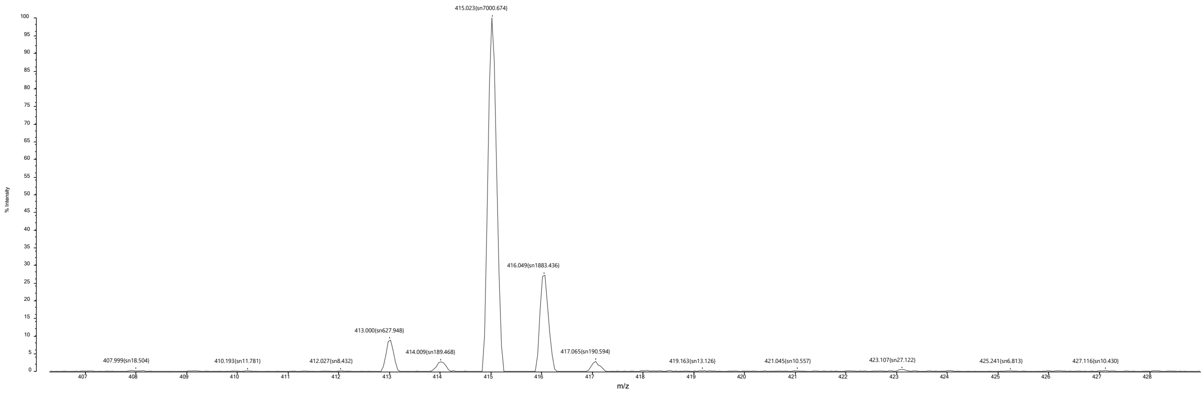

Figure S33. MALDI mass spectrum (with CHCA matrix) of Rh@TiO_2.

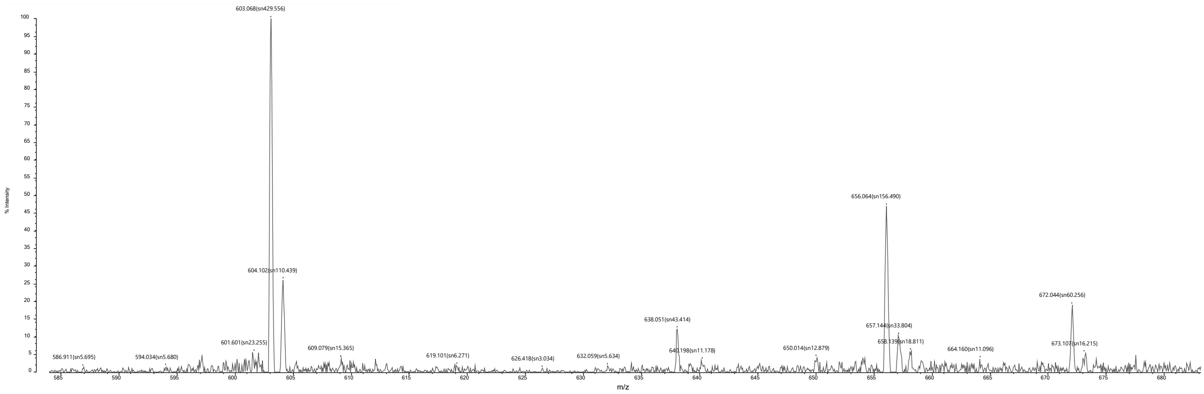

Figure S34. MALDI mass spectrum (with CHCA matrix) of Rh@TiO_2.

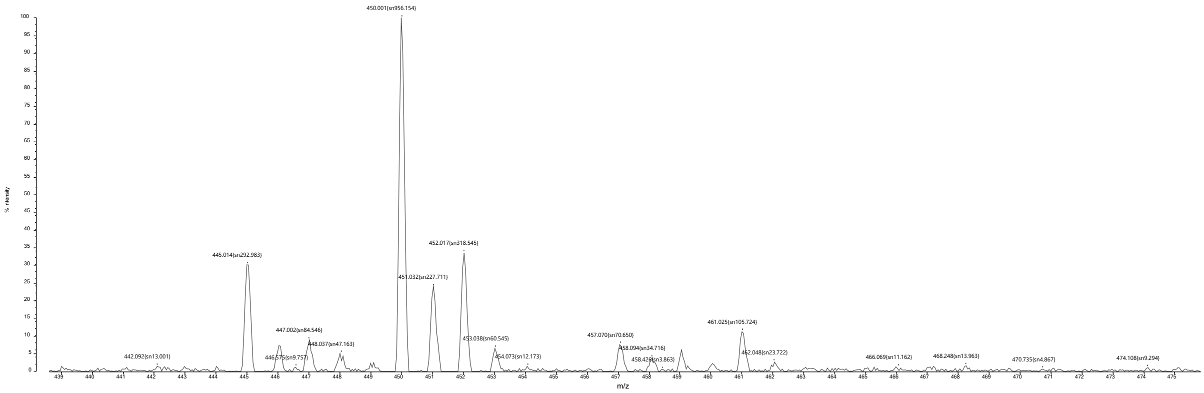

Figure S35. MALDI mass spectrum (with CHCA matrix) of Rh@TiO_2.

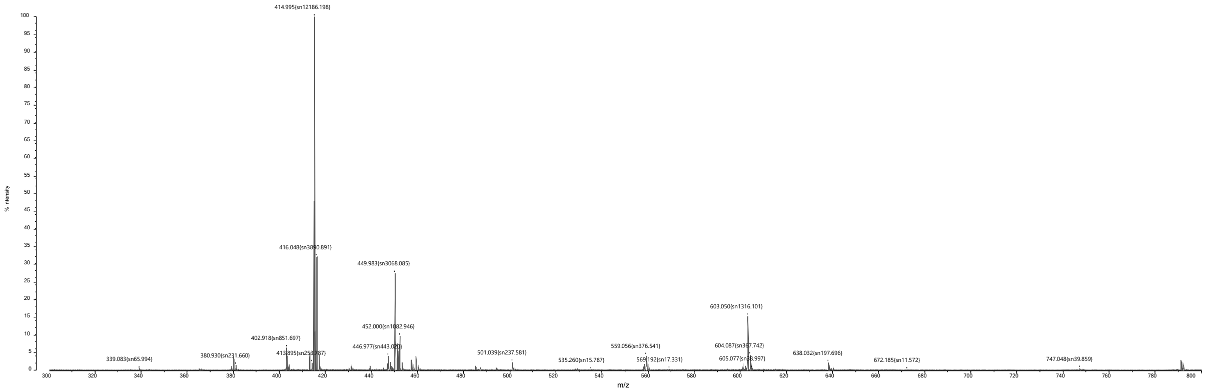

Figure S36. MALDI mass spectrum (with CHCA matrix) of rR@TiO_2.

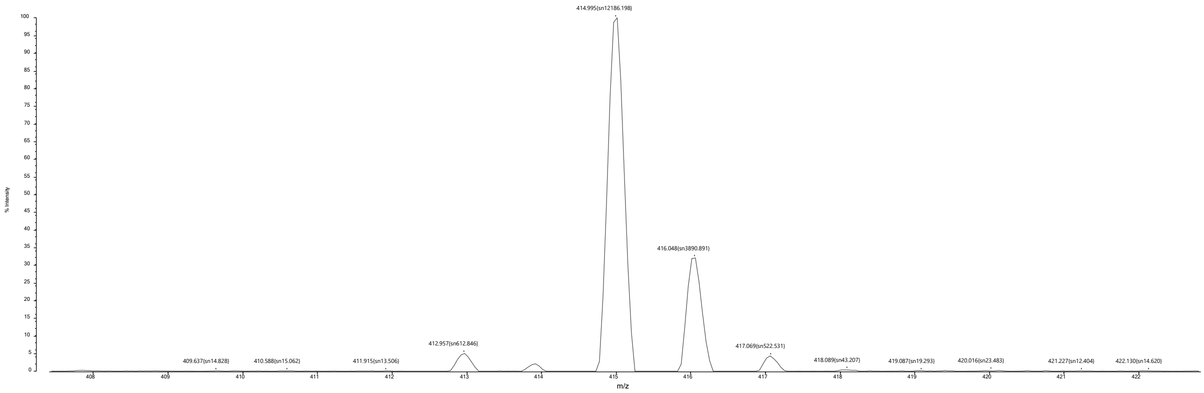

Figure S37. MALDI mass spectrum (with CHCA matrix) of rR@TiO_2.

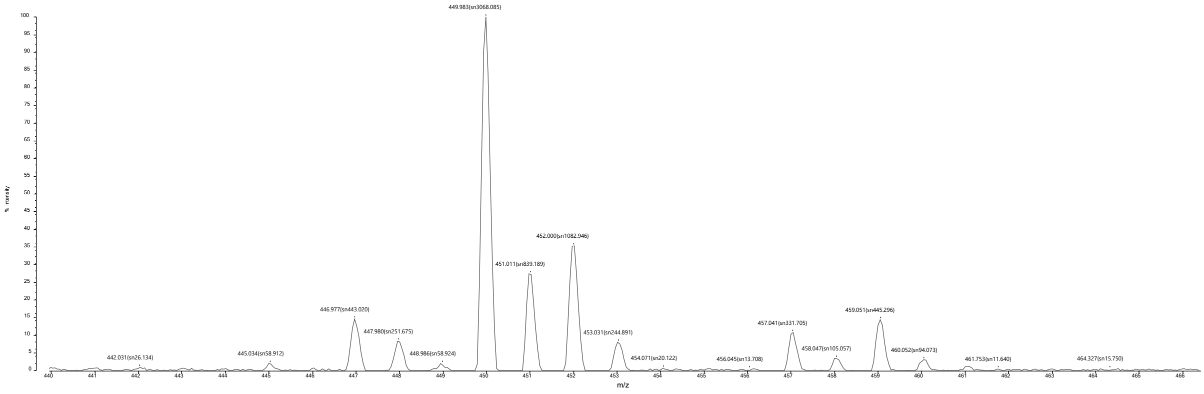

Figure S38. MALDI mass spectrum (with CHCA matrix) of rR@TiO_2.

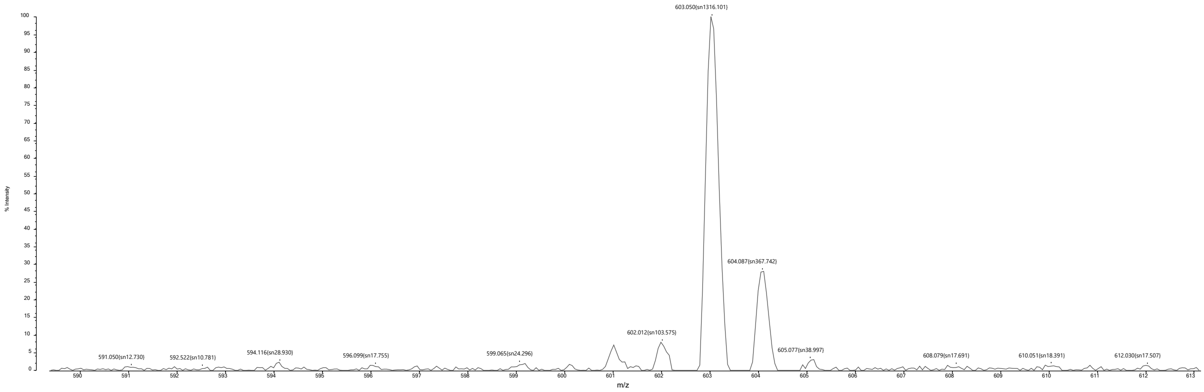

Figure S39. MALDI mass spectrum (with CHCA matrix) of rR@TiO_2.

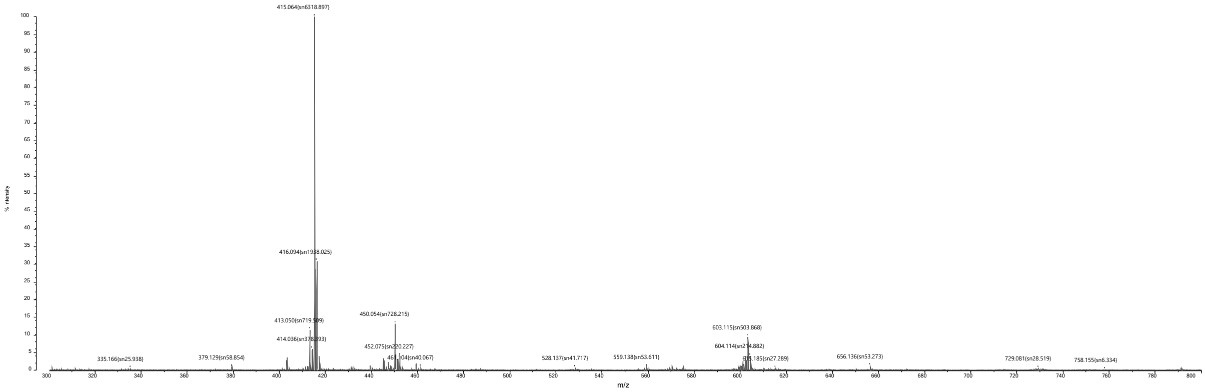

Figure S40. MALDI mass spectrum (with CHCA matrix) of RR@TiO_2.

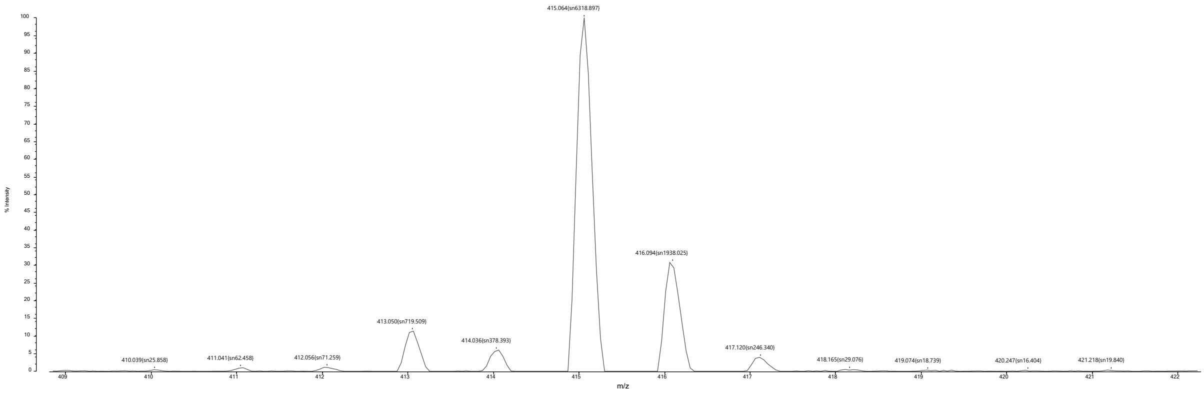

Figure S41. MALDI mass spectrum (with CHCA matrix) of RR@TiO_2.

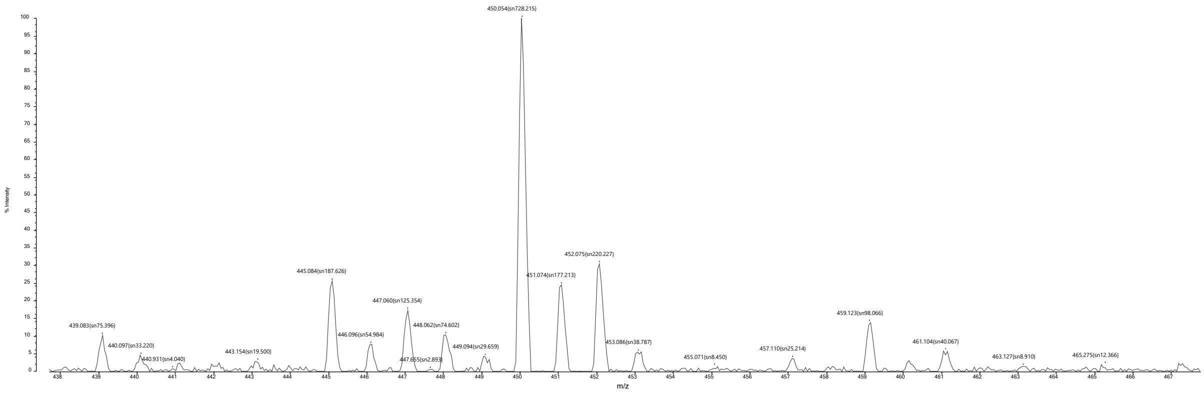

Figure S42. MALDI mass spectrum (with CHCA matrix) of RR@TiO_2.

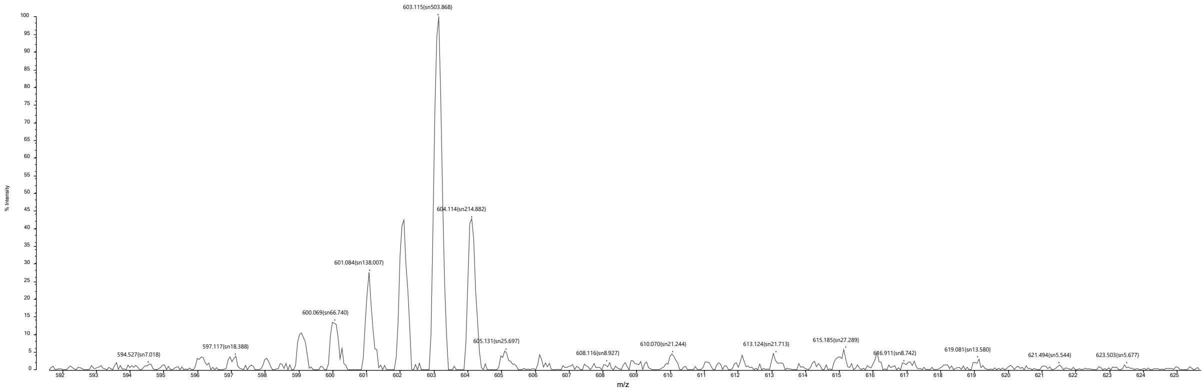

Figure S43. MALDI mass spectrum (with CHCA matrix) of RR@TiO_2.

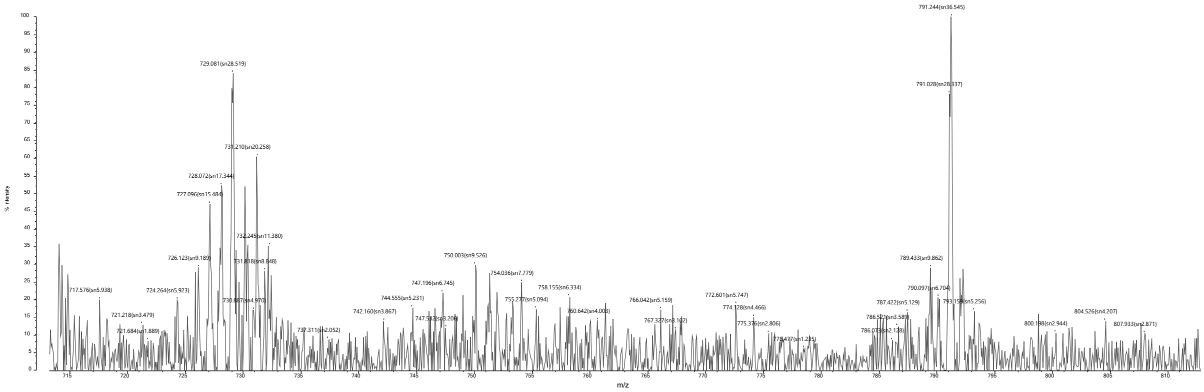

Figure S44. MALDI mass spectrum (with CHCA matrix) of RR@TiO_2.

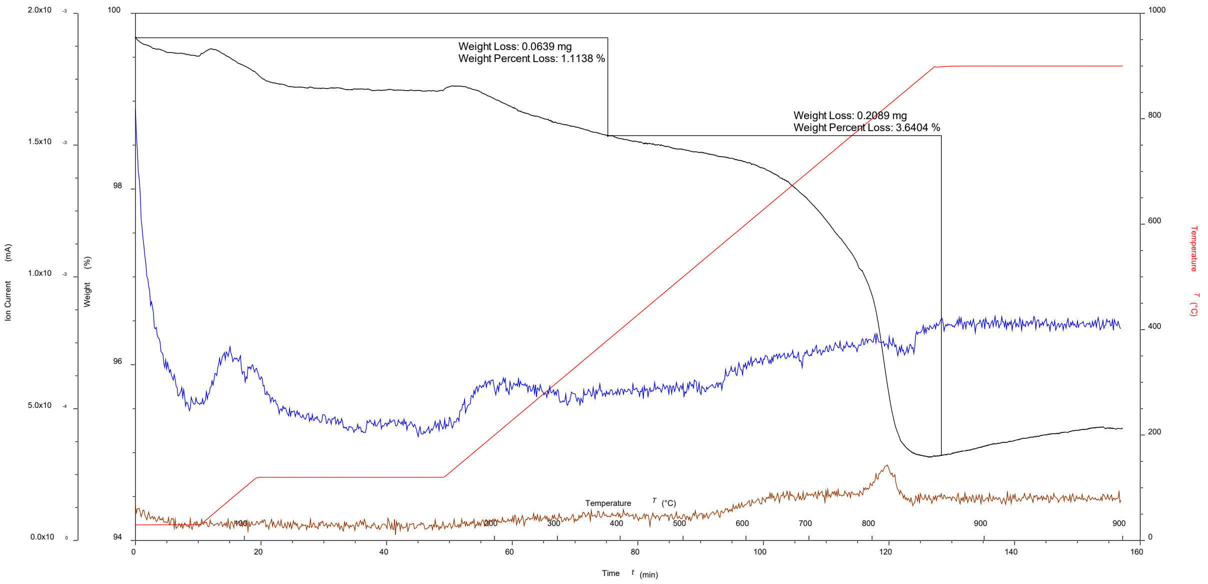

Figure S45. TGA curves for rR@TiO_2: Weight against time and temperature (blue), derivative of weight change against time (red).

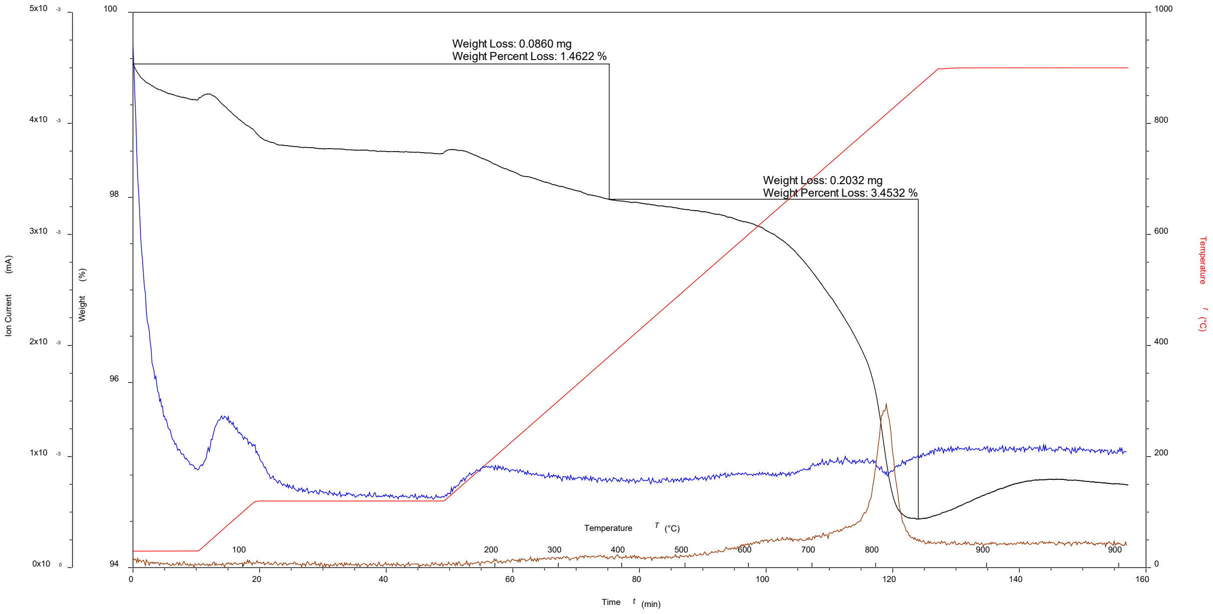

Figure S46. TGA curves for RR@TiO_2: Weight against time and temperature (blue), derivative of weight change against time (red).

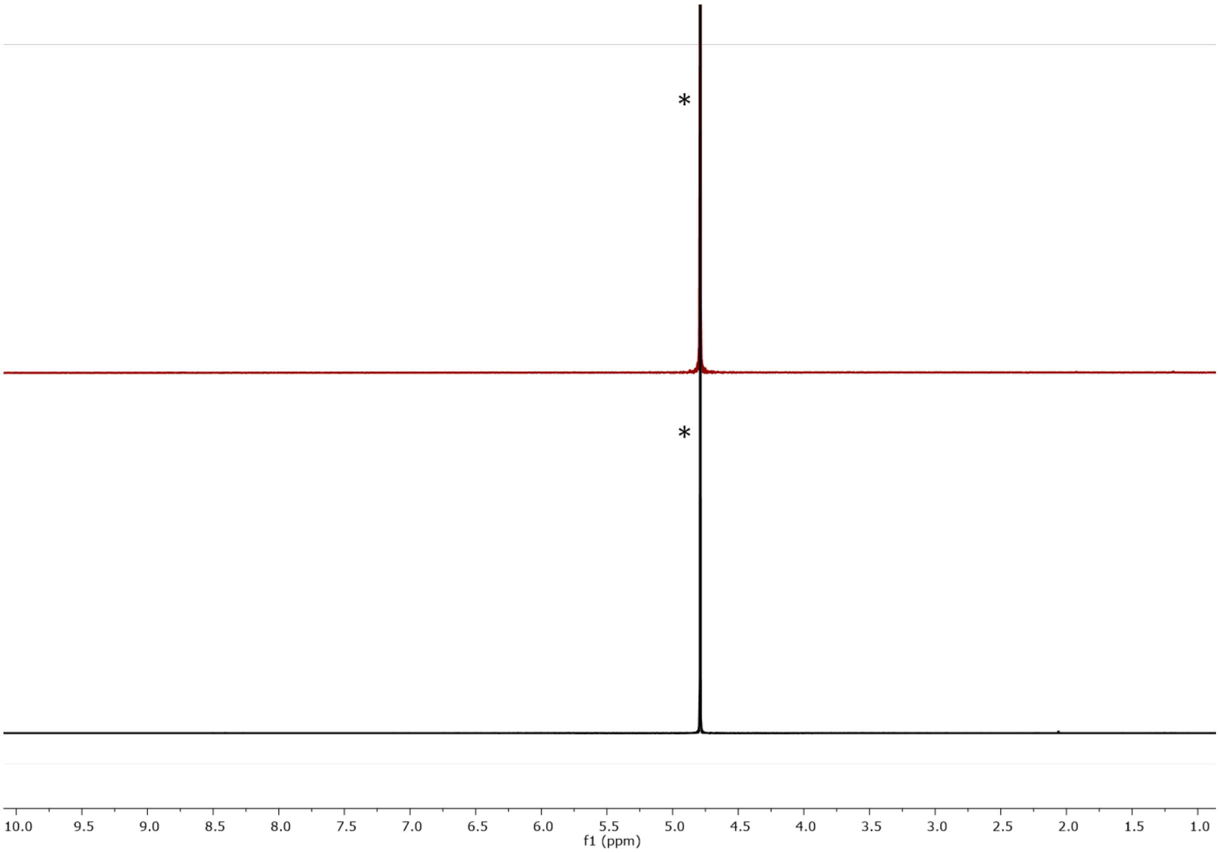

Figure S47. ^{1}H NMR (500 MHz, D_2O, 298 K) spectrum of rR@TiO_2 (black) and RR@TiO_2 (red), * = HOD. Chemical shifts in ⌀ /ppm.

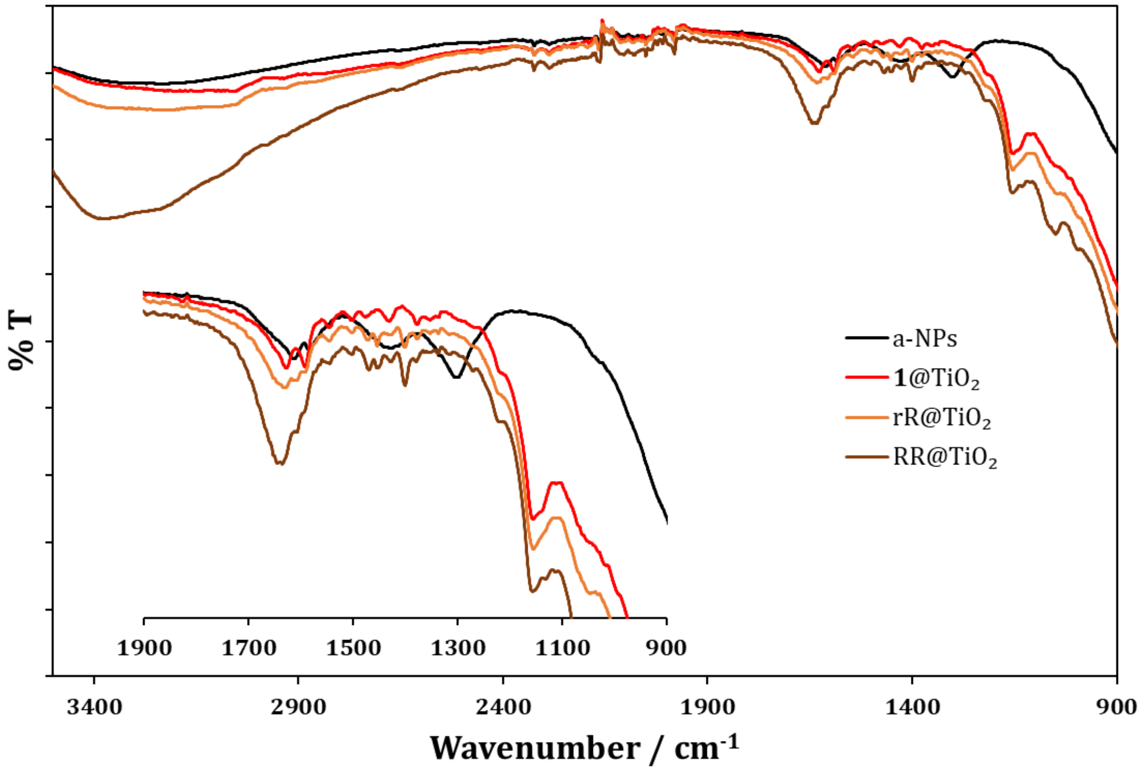

Figure S48. Solid-state IR spectra of a-NPs (black), **1**@TiO_2 (red), rR@TiO_2 (orange) and RR@TiO_2 (brown) with expansion of the low energy regions.

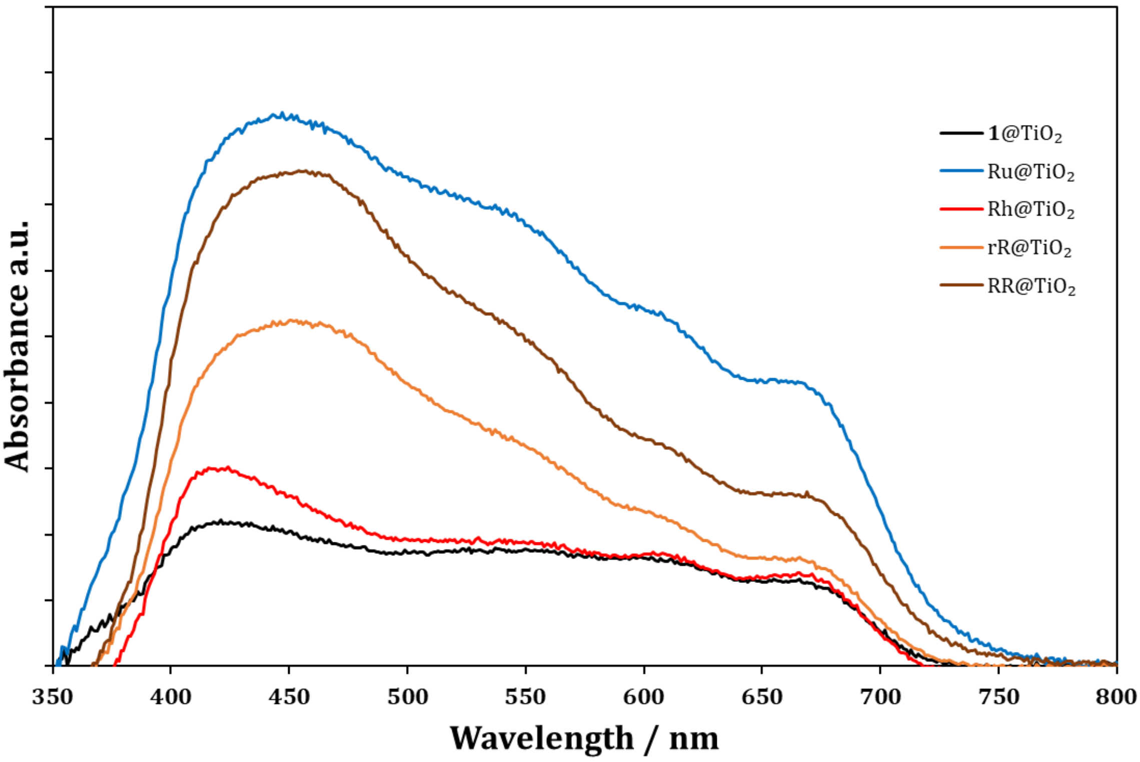

Figure S49. Solid-state absorption spectra of **1**@TiO_2 (black), Ru@TiO_2 (blue), Rh@TiO_2 (red), rR@TiO_2 (orange) and RR@TiO_2 (brown) using a-NP as reference.

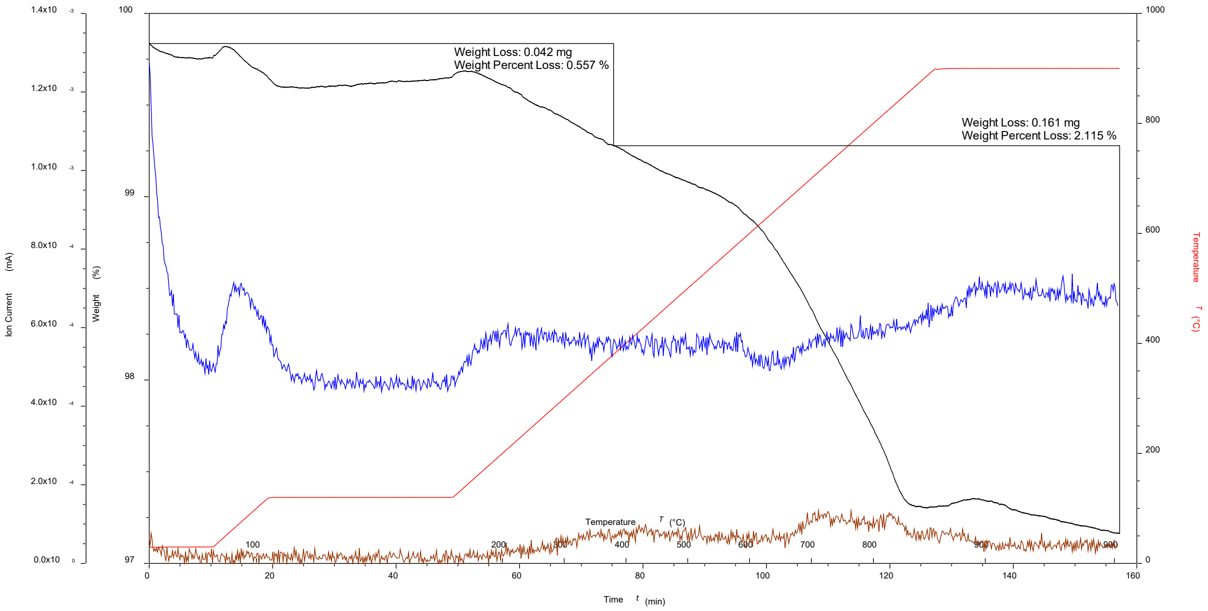

Figure S50. TGA curves for Ru@ZrO_2: Weight against time and temperature (blue), derivative of weight change against time (red).

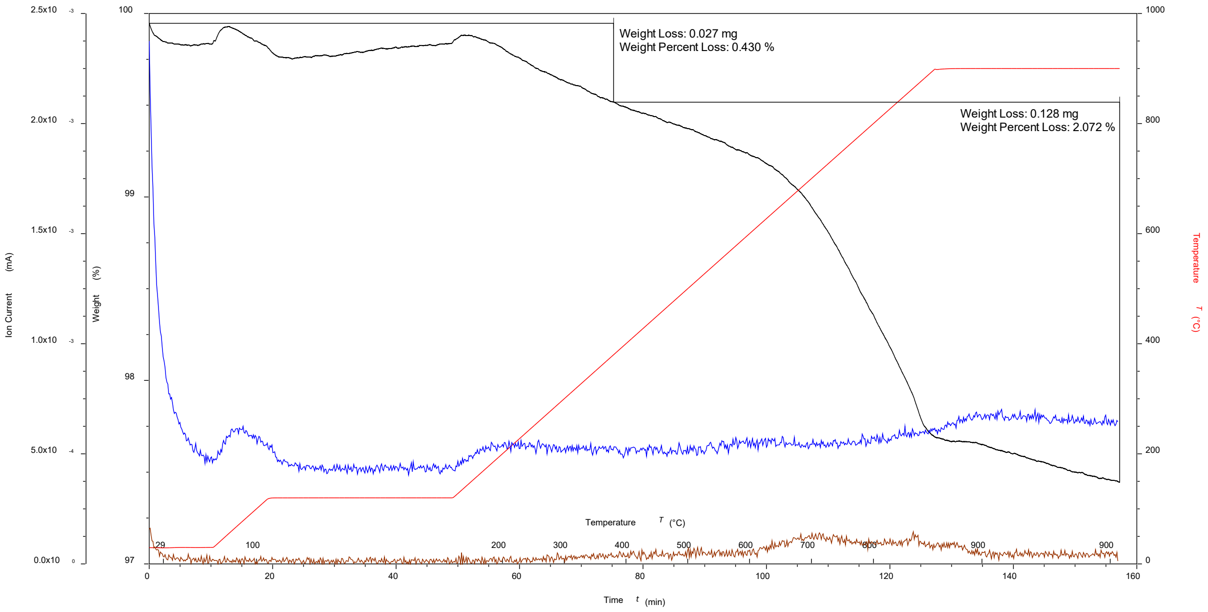

Figure S51. TGA curves for rR@ZrO2: Weight against time and temperature (blue), derivative of weight change against time (red).

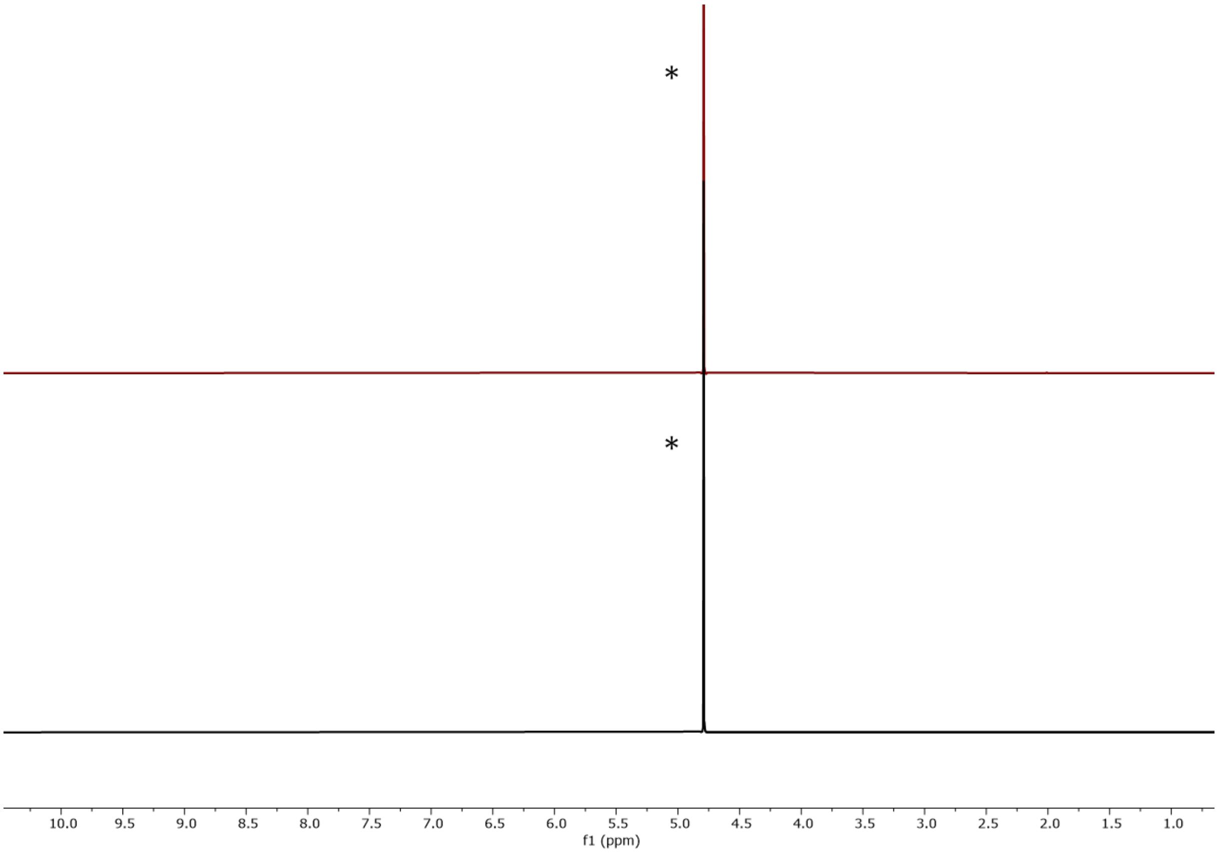

Figure S52. ^{1}H NMR (500 MHz, D_2O, 298 K) spectrum of rR@ZrO_2 (black) and RR@ZrO_2 (red), * = HOD. Chemical shifts in δ /ppm.

References

1. Zhang, D.; Telo, J.P.; Liao, C.; Hightower, S.E.; Clennan, E.L. Experimental and Computational Studies of Nuclear Substituted 1,1′-Dimethyl-2,2′-Bipyridinium Tetrafluoroborates. *J. Phys. Chem. A* **2007**, *111*, 13567-13574, doi:10.1021/jp074323u.
2. Maerker, G.; Case, F.H. The Synthesis of Some 4,4'-Disubstituted 2,2'-Bipyridines1. *J. Am. Chem. Soc.* **1958**, *80*, 2745-2748, doi:10.1021/ja01544a042.
3. Han, W.-S.; Han, J.-K.; Kim, H.-Y.; Choi, M.J.; Kang, Y.-S.; Pac, C.; Kang, S.O. Electronic Optimization of Heteroleptic Ru(II) Bipyridine Complexes by Remote Substituents: Synthesis, Characterization, and Application to Dye-Sensitized Solar Cells. *Inorg. Chem.* **2011**, *50*, 3271-3280, doi:10.1021/ic101909e.
4. Norris, M.R.; Concepcion, J.J.; Glasson, C.R.K.; Fang, Z.; Lapides, A.M.; Ashford, D.L.; Templeton, J.L.; Meyer, T.J. Synthesis of Phosphonic Acid Derivatized Bipyridine Ligands and Their Ruthenium Complexes. *Inorg. Chem.* **2013**, *52*, 12492-12501, doi:10.1021/ic4014976.
5. Montalti, M.; Wadhwa, S.; Kim, W.Y.; Kipp, R.A.; Schmehl, R.H. Luminescent Ruthenium(II) Bipyridyl−Phosphonic Acid Complexes: pH Dependent Photophysical Behavior and Quenching with Divalent Metal Ions. *Inorg. Chem.* **2000**, *39*, 76-84, doi:10.1021/ic991143t.
6. Freimann, S.A.; Prescimone, A.; Housecroft, C.E.; Constable, E.C. Turning over on sticky balls: preparation and catalytic studies of surface-functionalized TiO2 nanoparticles. *RSC Adv.* **2021**, *11*, 5537-5547, doi:10.1039/D0RA09319J.
